# Dupilumab efficacy and safety in patients with moderate to severe asthma: A systematic review and meta-analysis

**DOI:** 10.3389/fphar.2022.992731

**Published:** 2022-10-03

**Authors:** Mohamed Sayed Zaazouee, Asmaa Gomaa Alwarraqi, Yasmine Adel Mohammed, Mohamed A. Badheeb, Abdullah Mohamed Farhat, Mohammed Eleyan, Afnan Morad, Marwa Abdel-Aziz Zeid, Aya Shaban Mohamed, Hazem AbuEl-Enien, Ahmed Abdelalim, Ahmed Bostamy Elsnhory, Yasmin S. M. Hrizat, Nagat Taha Altahir, Doaa Atef, Alaa Ahmed Elshanbary, Khalaf F. Alsharif, Khalid J. Alzahrani, Mohammad Algahtani, Abdulrahman Theyab, Yousef M. Hawsawi, Ahmed A. Aldarmahi, Mohamed M. Abdel-Daim

**Affiliations:** ^1^ Faculty of Medicine, Al‐Azhar University, Assiut, Egypt; ^2^ Faculty of Medicine, Alexandria University, Alexandria, Egypt; ^3^ Faculty of Medicine, Assiut University, Assiut, Egypt; ^4^ College of Medicine, Hadhramout University, Al-Mukalla, Yemen; ^5^ Faculty of Medicine, Fayoum University, Fayoum, Egypt; ^6^ Department of Laboratory Medical Sciences, Alaqsa University, Gaza, Palestine; ^7^ Faculty of Medicine, Al-Azhar University, Gaza, Palestine; ^8^ Clinical Pharmacist, Pediatrics Department, Ministry of Health, Qalyubia, Egypt; ^9^ Faculty of Pharmacy, October 6 University, Giza, Egypt; ^10^ Department of Pharmacology, Faculty of Medicine, Assiut University, Assiut, Egypt; ^11^ Faculty of Pharmacy and Drug Manufacturing, Pharos University, Alexandria, Egypt; ^12^ Faculty of Pharmacy, Al-Azhar University, Cairo, Egypt; ^13^ Faculty of Medicine, Al-Azhar University, Cairo, Egypt; ^14^ Faculty of Medicine, University of Jordan, Amman, Jordan; ^15^ Faculty of Nursing, Khartoum University, Khartoum, Sudan; ^16^ Department of Emergency Medicine, Assiut University, Assiut, Egypt; ^17^ Department of Clinical Laboratory Sciences, College of Applied Medical Sciences, Taif University, Taif, Saudi Arabia; ^18^ Department of Laboratory and Blood Bank, Security Forces Hospital, Mecca, Saudi Arabia; ^19^ College of Medicine, Al-Faisal University, Riyadh, Saudi Arabia; ^20^ Research Center, King Faisal Specialist Hospital and Research Center, Jeddah, Saudi Arabia; ^21^ Basic Science Department, College of Science and Health Professions, King Saud Bin Abdulaziz University for Health Sciences, National Guard-Health Affairs, Jeddah, Saudi Arabia; ^22^ Department of Pharmaceutical Sciences, Pharmacy Program, Batterjee Medical College, Jeddah, Saudi Arabia; ^23^ Pharmacology Department, Faculty of Veterinary Medicine, Suez Canal University, Ismailia, Egypt

**Keywords:** asthma, dupilumab, monoclonal antibody, systematic review, meta-analysis

## Abstract

**Background:** Dupilumab is a human monoclonal antibody directed against the alpha subunit of the interleukin-4 receptor and inhibits the signaling of IL-4 and IL-13. It is approved for treating asthma and other type-2 inflammatory diseases. There is a conflict in the literature regarding the safety and efficacy of dupilumab. Thus, we aimed to assess the safety and efficacy of dupilumab in patients with moderate to severe asthma.

**Methods:** Six databases (PubMed, Embase, Scopus, Web of Science, Cochrane library, and clinicaltrials.gov registry) were searched until January 2022. We included randomized controlled trials that compared dupilumab with the placebo in moderate to severe asthma patients. We extracted the data at 12 and 24 weeks and analyzed them using review manager 5.4.

**Findings:** Thirteen trials were included. Dupilumab significantly improved the forced expiratory volume in 1 s, asthma control questionnaire score, the fraction of exhaled nitric oxide level, and immunoglobulin E level at 12 and 24 weeks (*p* < 0.05). However, it was associated with increased blood eosinophils at 12 and 24 weeks. Dupilumab was generally a safe agent for asthmatic patients. It showed no significant difference compared with the placebo regarding most adverse events.

**Conclusion:** Dupilumab improves pulmonary function and reduces local and systemic inflammatory markers with minimal adverse events in patients with moderate to severe asthma.

## 1 Introduction

Worldwide, asthma affected approximately 262 million people and caused 461,000 deaths (JL Murray, 2020). It is a major non-communicable disease that affects children and adults of both sexes, with a higher incidence in females (Wu et al., 2019). The disease prevalence has both genetic and environmental factors (Arrieta et al., 2015; Chen et al., 2017). Despite high-dose treatment, nearly more than 25% of the patients have uncontrolled asthma (JL Murray, 2020). In addition, those patients are at increased risk for respiratory function impairment, frequent asthmatic exacerbation, hospitalization, medical and societal costs, and poor quality of life (Bellin et al., 2015; Fleming et al., 2015).

Bronchial asthma is a disease of the air conducting system. It is characterized by a long-term airway inflammatory process even if the patient is in an asymptomatic period (Robinson et al., 1992). Major symptoms include cough, chest tightness, shortness of breath, and reversible episodic wheezes resulting from airway inflammation and hyperresponsiveness (Wu et al., 2019). The inflammatory process of asthma is mediated by helper T-2 cells and eosinophils in addition to the released cytokines, including interleukins (IL); IL-4, IL-5, and IL-13 (Robinson et al., 1992; Fahy, 2015). Interleukin-4 (IL-4) is one of the most important pro-inflammatory mediators in asthma. It mediates essential functions in asthma, including induction of the IgE isotype switch, expression of vascular cell adhesion molecule-1 (VCAM-1), and promotion of eosinophil transmigration across the endothelium, mucus secretion, and differentiation of T helper type-2 lymphocytes leading to cytokine release which causes asthma symptoms (Steinke and Borish, 2001). So, inhibiting the main ILs as IL-4 receptors will reduce the signaling and activity of the asthma inflammatory process, enhancing the pulmonary function and reducing the systemic and local inflammatory mediators.

Traditional pharmacological treatments are classified into controller medication and rescue medication. This comprises long-acting beta-agonists (LABA), inhaled corticosteroids, or leukotriene modifiers that interfere with the inflammatory process and prevent progression into irreversible airway remodeling (Newman, 2004; Chauhan and Ducharme, 2014; Wu et al., 2019). Dupilumab is a human monoclonal antibody directed against the alpha subunit of the interleukin-4 receptor and inhibits the signaling of IL-4 and IL-13 (Le Floc’h et al., 2020). The literature revealed significant improvement in clinical outcomes of asthmatic patients (Castro et al., 2018; Bachert et al., 2019; Castro et al., 2020; Bacharier et al., 2021). The effect of dupilumab starts early after the beginning of the treatment course. Most studies reported that it is maintained to the end of the follow-up periods of different RCTs up to 52 weeks. Moreover, it is approved for treating asthma and other type-2 inflammatory diseases in adults and adolescents. The global initiative for asthma (GINA) 2022 report (Reddel et al., 2022) suggests using anti-IL-4 receptors such as dupilumab in the management of patients with severe eosinophilic/type-2 asthma (step 5). This is suitable for patients of ≥6 years old, adolescents, and adults. However, other literature works revealed discrepancies regarding its efficacy (Wenzel et al., 2016; Weinstein et al., 2018; Laidlaw et al., 2021; Wechsler et al., 2021). This may be explained by different dosage regimens or comorbidities with asthma. Hence, in this systematic review and meta-analysis, we aimed to solve this contrast by evaluating the safety and efficacy of dupilumab in patients with moderate to severe asthma.

## 2 Materials and methods

### 2.1 Study design

We performed this systematic review and meta-analysis according to the Preferred Reporting Items for Systematic Reviews and Meta-Analysis (PRISMA) guidelines (Page et al., 2021) and the Cochrane Handbook of Systematic Review and Meta-analysis of Interventions (Higgins, 2019).

### 2.2 Search strategy

Six databases (PubMed, Embase, Scopus, Web of Science, Cochrane library, and clinicaltrials.gov registry) were used for literature search from inception until January 2022. We used the following keywords (Dupilumab, SAR231893, SAR-231893, Dupixent, REGN668, REGN-668, and Asthma*).

### 2.3 Inclusion and exclusion criteria

Human-based, English-written randomized controlled trials (RCTs) were included with no restriction on age, sex, settings, or publication dates. The included RCTs compared dupilumab with the placebo in moderate to severe asthma patients. Exclusion criteria included protocols, non-English–written studies, conference abstracts, book chapters, review articles, observational studies, and non-human studies.

### 2.4 Study selection and data extraction

We used the EndNote X8 version for citation management and duplicate removal. The full text of the eligible studies in the non-open access journals were obtained through academic institution access or by contacting authors requesting full texts of their studies.

The authors selected the studies according to two steps; first, we performed the title and abstract screening, and second, full-text screening to identify studies that fulfill our inclusion criteria. We manually screened the reference list in the included studies and citations of the identified articles. Four independent authors performed each step, and a discussion with the supervisor solved any disagreements.

Four authors extracted the following data (I) summary of included studies, including study design, NCT numbers, participants’ details, intervention period, follow-up period, primary outcomes, and (II) baseline characteristics of included studies, including study arms, sample size, age, sex, forced expiratory volume in 1 s (FEV1) reversibility, history of nasal polyposis, history of smoking, and allergic conditions. Another three authors extracted the outcomes of interest.

### 2.5 Outcomes

#### 2.5.1 Primary outcomes

##### 2.5.1.1 Efficacy outcomes

FEV1 change per liter, Asthma Control Questionnaire (ACQ) change.

##### 2.5.1.2 Safety outcomes

Any treatment-emergent adverse events, any treatment-emergent adverse events leading to permanent discontinuation.

#### 2.5.2 Secondary outcomes

##### 2.5.2.1 Efficacy outcomes

Fraction of exhaled nitric oxide (FeNO) change, blood eosinophil change, and IgE changes all at 12 and 24 weeks.

##### 2.5.2.2 Safety outcomes

Any adverse events, any adverse events leading to permanent discontinuation, serious adverse events, serious treatment-emergent adverse events, any adverse events leading to death, any treatment-emergent adverse events leading to death, nasopharyngitis, upper respiratory tract infection, viral upper respiratory tract infection, headache, erythema, injection-site reaction, cough, allergic rhinitis, bronchitis, influenza, urinary tract infection, back pain, sinusitis, and eosinophilia.

### 2.6 Quality assessment

The risk of bias was assessed according to the Cochrane risk of bias tool, using the Cochrane Handbook for Systematic Reviews of Interventions 5.1.0 (Higgins et al., 2011). It includes seven main domains, namely, random sequence generation, allocation sequence concealment, blinding of participants and personnel, blinding of outcome assessment, incomplete outcome data, selective outcome reporting, and other biases.

### 2.7 Statistical analysis

We used Review Manager (Version 5.4) to analyze the data. We used the risk ratio (RR) with a 95% confidence interval (CI) for dichotomous data and mean difference (MD) and 95% CI for continuous data. The data were pooled under a random-effect model. Heterogeneity among the studies was examined using Cochrane’s *p* values and I^2^. We considered the data heterogeneous when chi-square *p* < 0.1 and I^2^ >50%. We used a sensitivity analysis by leaving one out method to overcome heterogeneity. According to the Cochrane Handbook, we could not assess the publication bias as all outcomes were reported in less than 10 studies. The efficacy outcomes were pooled at different time points, 12 and 24 weeks. In addition, we performed a subgroup analysis according to the treatment regimen. This includes the following groups: 100–200 mg of dupilumab every 2 weeks, 200 mg dupilumab every 2 weeks, 200 mg dupilumab every 4 weeks, 300 mg dupilumab every 2 weeks, and 300 mg dupilumab every 4 weeks.

## 3 Results

### 3.1 Summary of study selection and general characteristics of included studies

**FIGURE 1:**
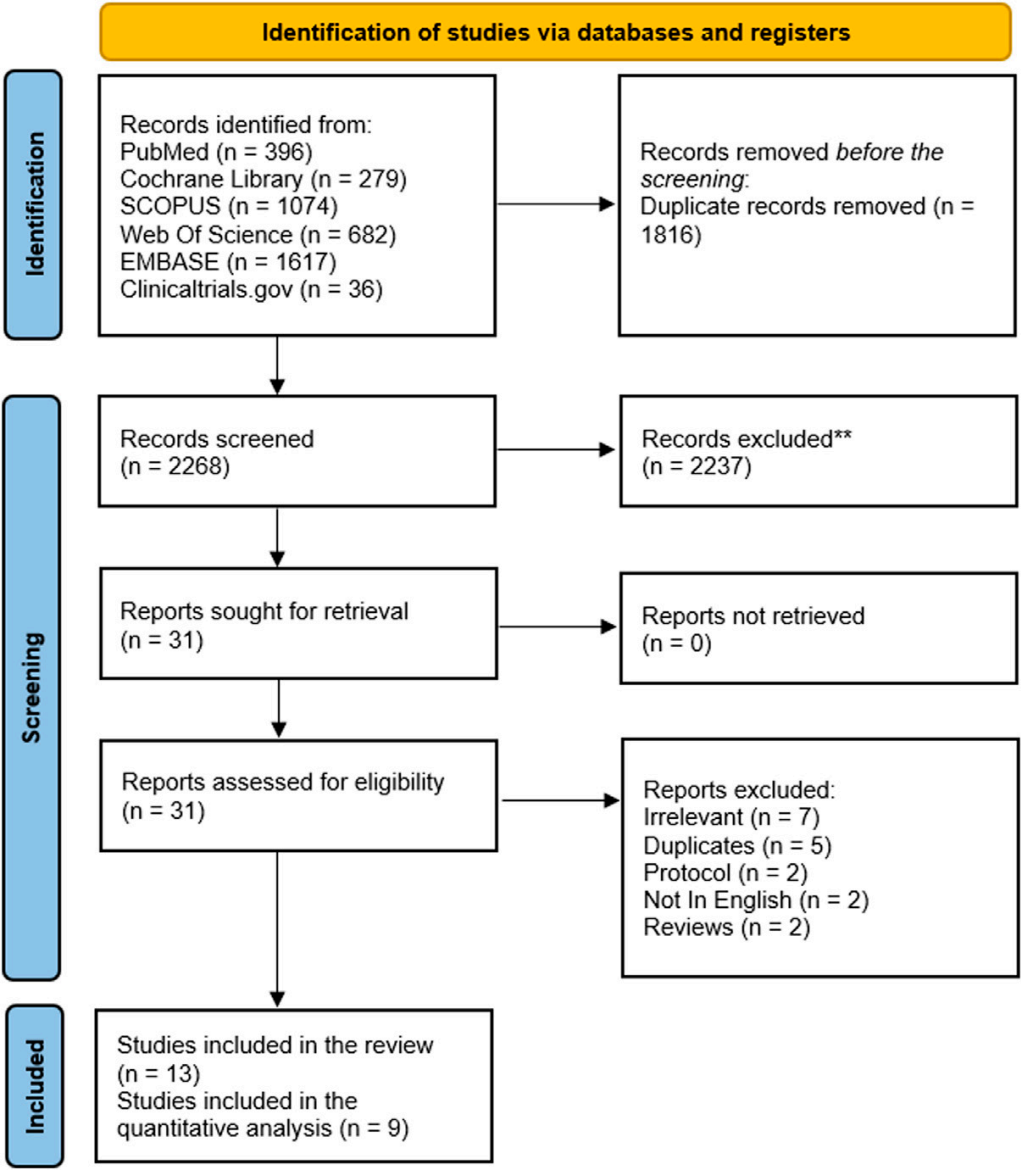
PRISMA flow diagram of the included studies.

A total of 2,268 studies were retrieved from different databases after duplicate removal. Of them, only 31 studies were eligible for full-text assessment. According to our inclusion and exclusion criteria, we included 13 RCTs (Wenzel et al., 2013; Wenzel et al., 2016; Castro et al., 2018; Rabe et al., 2018; Weinstein et al., 2018; Bachert et al., 2019; Castro et al., 2020; Corren et al., 2020; Tohda et al., 2020; Bacharier et al., 2021; Corren et al., 2021; Laidlaw et al., 2021; Wechsler et al., 2021) in our systematic review; of them, nine trials were eligible for our meta-analysis (Wenzel et al., 2013; Wenzel et al., 2016; Castro et al., 2018; Rabe et al., 2018; Weinstein et al., 2018; Bachert et al., 2019; Bacharier et al., 2021; Laidlaw et al., 2021; Wechsler et al., 2021). Figure 1 shows the PRISMA flow diagram of our meta-analysis.

We included data of 4,482 patients. Of them, 726 (16.2%) were smokers. A total of 1,092 patients (24.4%) had a history of polyposis, while 2,558 patients (57%) had a history of allergic conditions. The mean age of our population was 45 years old. Most of them were females (59.2%). The mean FEV1 reversibility at baseline was 21.2%. The follow-up periods ranged from 12 to 52 weeks. Tables 1, 2 show the summary and baseline characteristics of the included population.

**TABLE 1:** Summary of the included studies.

Study ID	Study design	NCT	Participants details	Intervention period	Follow-up period	Primary outcomes
Bacharier et al. (2021)	RCT	NCT02948959	- Children of 6–11 years old	- 52 weeks	-	Percentage of predicted prebronchodilator forced expiratory volume in 1 s
			- Had moderate to severe asthma according to GINA guidelines	- After week 12, home administration was allowed		
Castro et al. (2018)	RCT	NCT02414854	- Patients of 12 years or older	52 weeks	12 weeks	The annualized rate of severe asthma exacerbations and the absolute forced expiratory volume in 1 s
			- Had uncontrolled, moderate to severe asthma for ≥1 year, according to GINA guidelines			
Castro et al. (2020)	Post-hoc analysis	NCT02414854	- Patients of 12 years or older	52 weeks	12 weeks	The annualized rate of severe asthma exacerbations and the absolute forced expiratory volume in 1 s
			- Had uncontrolled, moderate to severe asthma for ≥1 year, according to GINA guidelines			
Corren et al. (2019)	Post-hoc analysis	NCT02414854	- Patients of 12 years or older	52 weeks	12 weeks	The annualized rate of severe asthma exacerbations and the absolute forced expiratory volume in 1 s (according to allergic asthma presence)
			- Had uncontrolled, moderate to severe asthma for ≥1 year, according to GINA guidelines			
Corren et al. (2021)	Post-hoc analysis	NCT02414854	- Patients of 12 years or older	52 weeks	12 weeks	The annualized rate of severe asthma exacerbations and the absolute forced expiratory volume in 1 s (according to Eosinophil’s count)
			- Had uncontrolled, moderate to severe asthma for ≥1 year, according to GINA guidelines			
Tohda et al. (2020)	Post-hoc analysis	NCT02414854	- Japanese patients of 12 years or older	52 weeks	12 weeks	The annualized rate of severe asthma exacerbations and the absolute forced expiratory volume in 1 s (in Japanese only)
			- Had uncontrolled, moderate to severe asthma for ≥1 year, according to GINA guidelines			
Bachert et al. (2020)	RCT	- NCT02912468	- Adults of 18 years or older	24 weeks	24 weeks	Bilateral nasal polyp score and nasal congestion or obstruction score
			- Had CRSwNP and had corticosteroids for 2 years or previous sinonasal surgery			
		- NCT02898454	- 50% of these patients had asthma	52 weeks	12 weeks	
Laidlaw et al. (2021)	Post-hoc analysis	- NCT02912468	- Adults of 18 years or older	24 weeks	24 weeks	Bilateral nasal polyp score and nasal congestion or obstruction score. (Longer follow-up)
			- Had CRSwNP and had corticosteroids for 2 years or previous sinonasal surgery			
		- NCT02898454	- 50% of these patients had asthma	52 weeks	12 weeks	
Rape et al. (2018)	RCT	NCT02528214	- Patients of 12 years or older	24 weeks	12 weeks	Percentage reduction in the glucocorticoid dose
			- Had asthma for ≥1 year, according to GINA guidelines, and received glucocorticoids for 6 months			
Wechsler et al. (2021)	RCT	NCT03387852	- Patients of 18 to 70 years old	12 weeks	20 weeks	Event indicating a loss of asthma control
			- Had asthma for ≥1 year, according to GINA guidelines, and received glucocorticoids and LABA for ≥3 months			
Weinstein et al. (2018)	Post-hoc analysis	NCT01854047	- Adults of 18 years or older	24 weeks	16 weeks	Change in forced expiratory volume in 1 s according to perennial allergic rhinitis presence)
			- Had asthma for ≥1 year, according to GINA guidelines			
Wenzel et al. (2016)	RCT	NCT01854047	- Adults of 18 years or older	24 weeks	16 weeks	Change in forced expiratory volume in 1 s
			- Had asthma for ≥1 year, according to GINA guidelines			
Wenzel et al. (2013)	RCT	NCT01312961	- Patients of 18 to 65 years old	12 weeks	8 weeks	Occurrence of an asthma exacerbation
			- Had persistent, moderate-to-severe asthma for ≥1 year and had ≥300 cells/µl eosinophil in blood or ≥3% in sputum			

GINA; Global Initiative for Asthma, CRSwNP; chronic rhinosinusitis with nasal polyps, LABA; long-acting beta-agonist, RCT; randomized controlled trial.

**TABLE 2:** Baseline characteristics of the study population.

Study ID	Study arm	Sample	Age, year	Sex, male	FEV1 reversibility %	Nasal polyposis history	Former smoker	Allergic condition
Bacharier et al., 2021	Dupilumab 100–200 mg q2w	273	8.9 ± 1.7	175 (64.1)	21.56 ± 22.43	—	—	—
	Placebo	135	8.9 ± 1.6	87 (64.4)	15.63 ± 16.33	—	—	—
Castro et al., 2018, Castro et al., 2020, Corren et al., 2019, Corren et al., 2021	Dupilumab 300 mg q2w	633	47.7 ± 15.6	239 (37.8)	26.29 ± 21.73	145 (22.9)	116 (18.3)	524 (82.8)
	Placebo	321	48.2 ± 14.7	103 (32.1)	25.73 ± 17.65	80 (24.9)	67 (20.9)	266 (82.9)
	Dupilumab 200 mg q2w	631	47.9 ± 15.3	244 (38.7)	27.39 ± 22.79	141 (22.3)	126 (20.0)	509 (80.7)
	Placebo	317	48.2 ± 15.6	119 (37.5)	25.06 ± 18.76	73 (23.0)	59 (18.6)	266 (83.9)
Laidlaw et al., 2021 and Bachert et al., 2020	Dupilumab 300 mg q2w	258	34.78 ± 16.01	210 (49.1)	—	428 (100)	—	—
	Placebo	170			—		—	—
Rape et al., 2018	Dupilumab 300 mg q2w	103	51.9 ± 12.5	41 (40)	—	33 (32)	24 (23)	10 (10)
	Placebo	107	50.7 ± 12.8	42 (39)	—	38 (36)	17 (16)	10 (9)
Tohda et al., 2020	Dupilumab 300 mg q2w	41	47.2 ± 18.2	13 (31.7)	20.11 ± 17.54	8 (19.5)	4 (9.8)	36 (87.8)
	Placebo	17	51.4 ± 12.9	5 (29.4)	21.55 ± 17.95	7 (41.2)	6 (35.3)	13 (76.5)
	Dupilumab 200 mg q2w	37	49 ± 16	19 (51.4)	20.63 ± 19.64	12 (32.4)	10 (27.0)	33 (89.2)
	Placebo	19	47.1 ± 16.9	8 (42.1)	21 ± 11.44	2 (10.5)	6 (31.6)	18 (94.7)
Wechsler et al., 2021	Dupilumab 300 mg	75	51.3 ± 12.7	34 (45)	13.32 ± 11.76	—	14 (19)	66 (88)
	Placebo	74	47 ± 11.4	27 (36)	15.58 ± 15.84	—	17 (9)	67 (91)
Weinstein et al. 2018- with PAR	Dupilumab 300 mg q2w	84	45 ± 13.2	34 (40.5)	—	—	20 (23.8)	53 (63.9)
	Dupilumab 200 mg q2w	73	46.6 ± 14.6	24 (32.9)	—	—	17 (23.3)	54 (75)
	Placebo	84	47.9 ± 12.9	34 (40.5)	—	—	18 (21.4)	59 (71.1)
Weinstein et al. 2018- without PAR	Dupilumab 300 mg q2w	43	48.8 ± 11.5	8 (18.6)	—	—	9 (20.9)	21 (51.2)
	Dupilumab 200 mg q2w	52	55.6 ± 10.5	14 (26.9)	—	—	9 (17.3)	29 (55.8)
	Placebo	56	51.9 ± 13.2	12 (21.4)	—	—	13 (23.2)	30 (56.6)
Wenzel et al., 2013	Dupilumab 300 mg q2w	52	37.8 ± 13.2	26 (50)	—	—	—	—
	Placebo	52	41.6 ± 13.1	26 (50)	—	—	—	—
Wenzel et al., 2016	Dupilumab 200 mg q4w	154	47.9 ± 13.1	67 (43·5)	—	21 (13·9)	34 (22·2)	100 (66·2)
	Dupilumab 300 mg q4w	157	47.9 ± 13.1	57 (36·3)	—	31 (20·0)	38 (24·2)	99 (63·9)
	Dupilumab 200 mg q2w	157	51 ± 13.4	54 (36·0)	—	25 (16·8)	32 (21·3)	99 (66·4)
	Dupilumab 300 mg q2w	150	47.5 ± 12.4	54 (34·4)	—	30 (19·5)	36 (22·9)	94 (61·0)
	Placebo	157	49 ± 12.7	54 (34·2)	—	18 (11·7)	34 (21·5)	102 (66·2)

PAR; perennial allergic rhinitis, FEV1; forced expiratory volume in 1 s. Data are reported as mean ± standard deviation or number (percentage).

### 3.2 Results of the quality assessment

**FIGURE 2:**
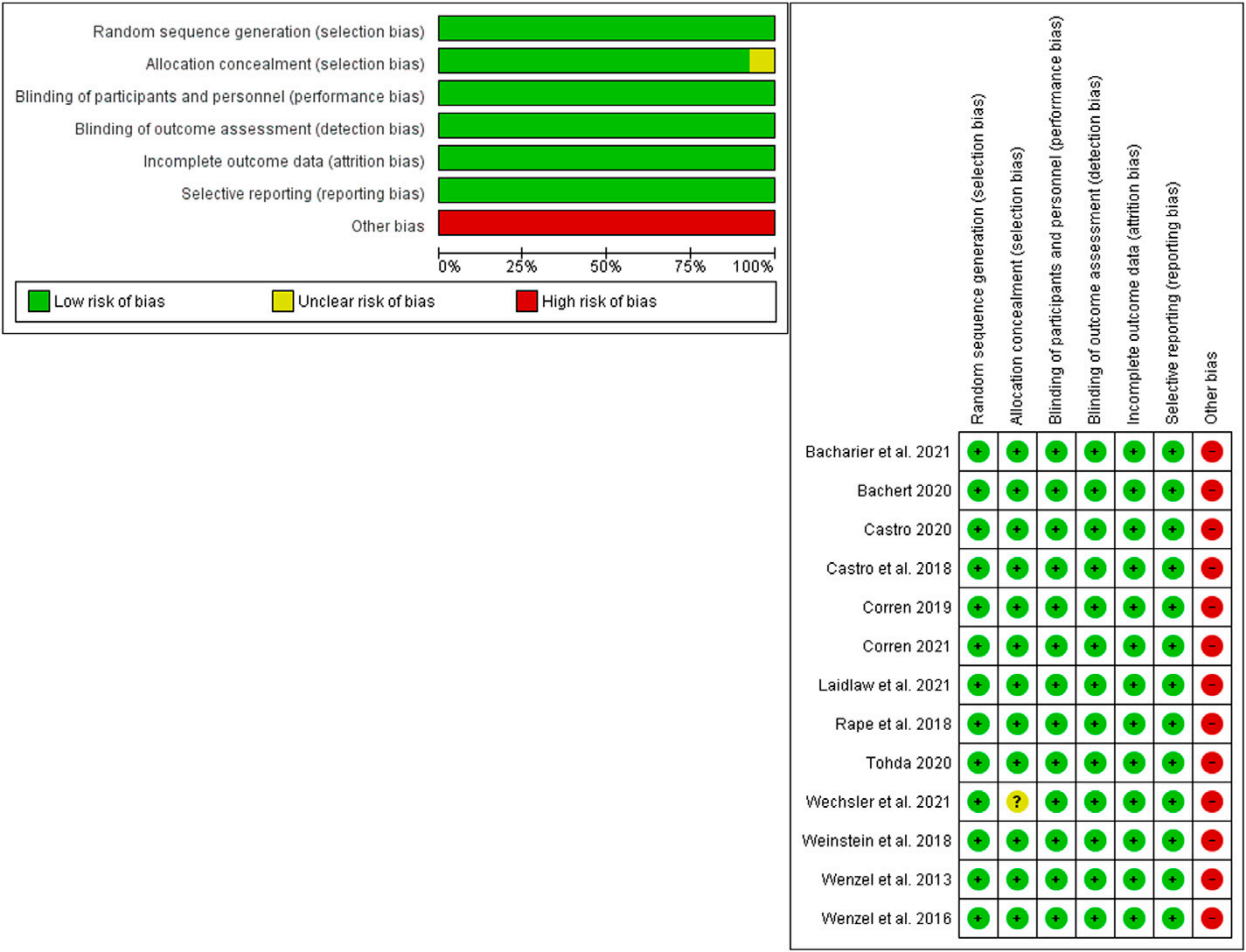
Summary and graph of risk of bias assessment results for the included studies.

All the included studies revealed a low risk of bias regarding all the assessed domains of the Cochrane risk of bias tool, except for other bias domains. It was put at a high risk as all of the included studies were funded by the drug manufacturer. In addition, in the trial by Wechesler et al. (Laidlaw et al., 2021), data about the allocation process were unclear. Figure 2 shows the summary of the quality assessment.

### 3.3 Analysis of the outcomes

#### 3.3.1 Change in clinical characteristics after 12 and 24 weeks

##### 3.3.1.1 Change in FEV1 (L) at week 12

**FIGURE 3:**
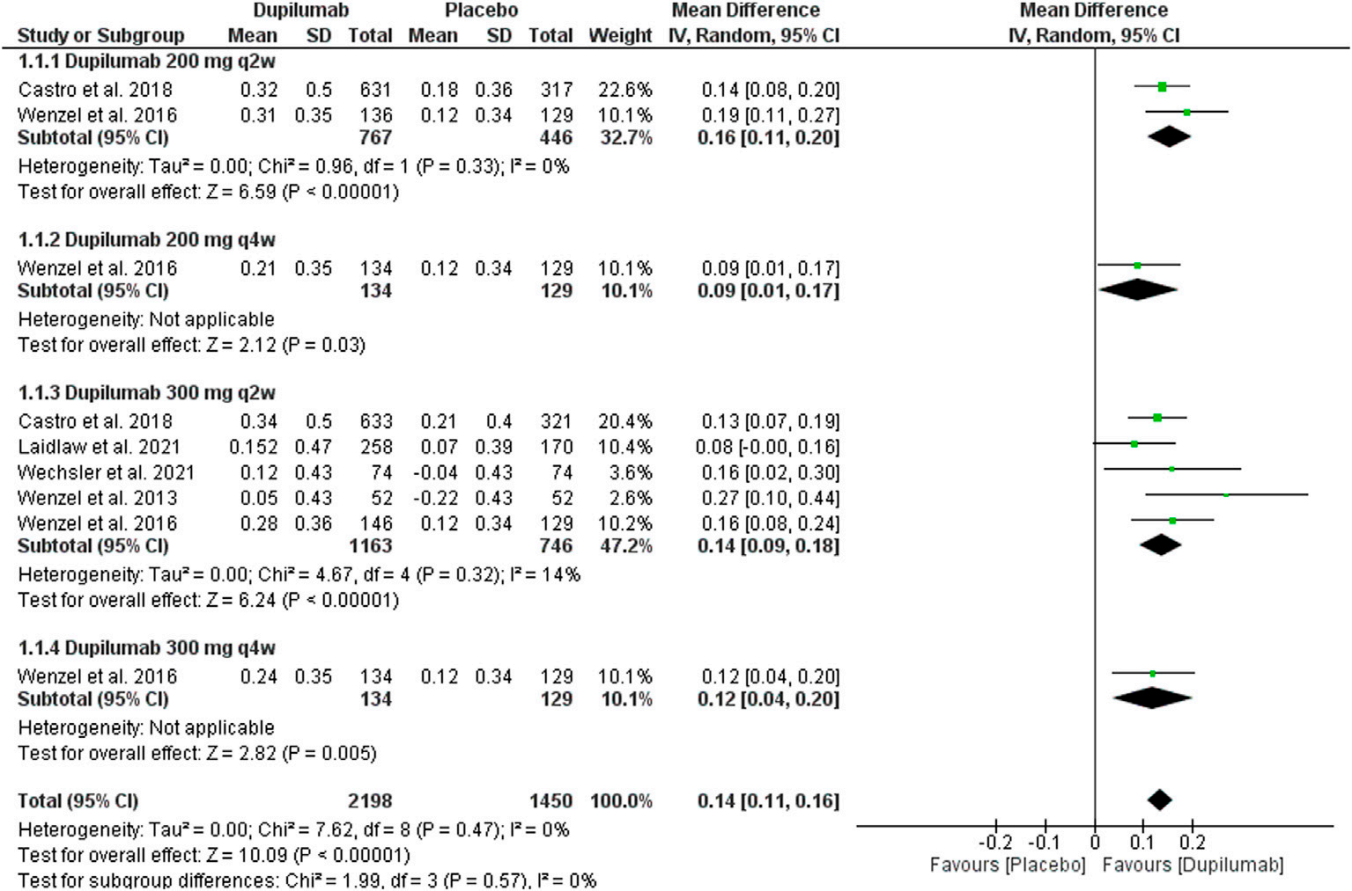
Results of the FEV1 change at the 12th week.

This outcome was reported by five trials (Wenzel et al., 2013; Wenzel et al., 2016; Castro et al., 2018; Laidlaw et al., 2021; Wechsler et al., 2021). Dupilumab significantly improved the absolute (dose-independent) FEV1 (L) at week 12 among 2,198 patients in this group compared with 1,450 patients in the placebo group; MD = 0.14, 95% CI = 0.11, 0.16, *p* < 0.01. This outcome was homogeneous *p* = 0.47, I^2^ = 0%. The subgroup analysis of different dupilumab doses did not reveal any significant difference between all subgroups *p* = 0.57 (Figure 3).

As for dupilumab 200 mg q2w, two trials reported this outcome (Wenzel et al., 2016; Castro et al., 2018). They revealed a significant improvement in FEV1 (L) compared with the placebo; MD = 0.16 (95% CI = 0.11, 0.20), *p* < 0.01. This subgroup pooled data were homogeneous; *p* = 0.33, I^2^ = 0%.

Regarding the 300 mg dupilumab q2w, five trials reported this outcome (Wenzel et al., 2013; Wenzel et al., 2016; Castro et al., 2018; Laidlaw et al., 2021; Wechsler et al., 2021). Dupilumab significantly improved the FEV1(L) compared with the placebo; MD = 0.14, 95% CI = 0.09, 0.18, *p* < 0.01. This subgroup pooled data were homogeneous; *p* = 0.32, I^2^ = 14%.

##### 3.3.1.2 Change in FEV1 (L) at week 24

**FIGURE 4:**
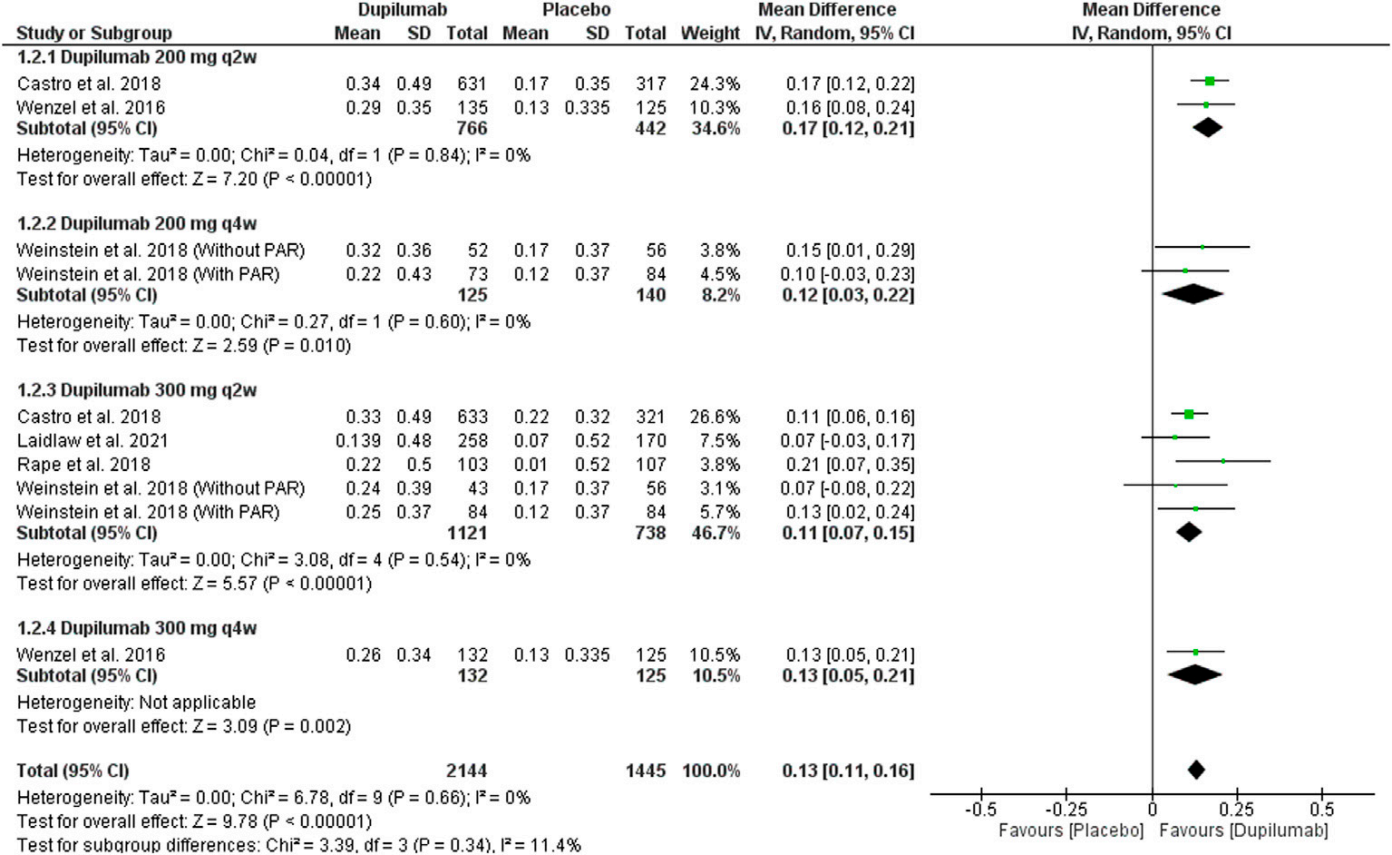
Results of the FEV1 change at the 24th week.

This outcome was reported by five trials (Wenzel et al., 2016; Castro et al., 2018; Rabe et al., 2018; Weinstein et al., 2018; Laidlaw et al., 2021). Dupilumab significantly improved the absolute (dose-independent) FEV1 (L) at week 24 among 2,144 patients in the treatment group compared with 1,445 patients in the placebo group; MD = 0.13, (95% CI = 0.11, 0.16), *p* < 0.00001. This outcome was homogeneous; *p* = 0.66, I^2^ = 0%. The subgroup analysis revealed no significant difference between them, *p* = 0.34 (Figure 4).

In the dupilumab 200 mg q2w subgroup, two included trials (Wenzel et al., 2016; Castro et al., 2018) revealed a significant improvement in FEV1 in the dupilumab group; MD = 0.17 (95% CI = 0.12, 0.21), *p* < 0.00001. This subgroup pooled data were homogeneous; *p* = 0.84, I^2^ = 0%.

As for 200 mg dupilumab q4w, it significantly improved the FEV1 compared with the placebo; MD = 0.12 (95% CI = 0.03, 0.22), *p* = 0.01. This subgroup data were homogeneous; *p* = 0.6, I^2^ = 0%.

In four trials (Castro et al., 2018; Rabe et al., 2018; Weinstein et al., 2018; Laidlaw et al., 2021), the dupilumab 300 mg q2w regimen revealed a significant improvement in FEV1 compared with the placebo; MD = 0.11 (95% CI = 0.07, 0.15), *p* < 0.00001. This subgroup data were homogeneous; *p* = 0.54, I^2^ = 0%.

##### 3.3.1.3 Change in ACQ at week 12

**FIGURE 5:**
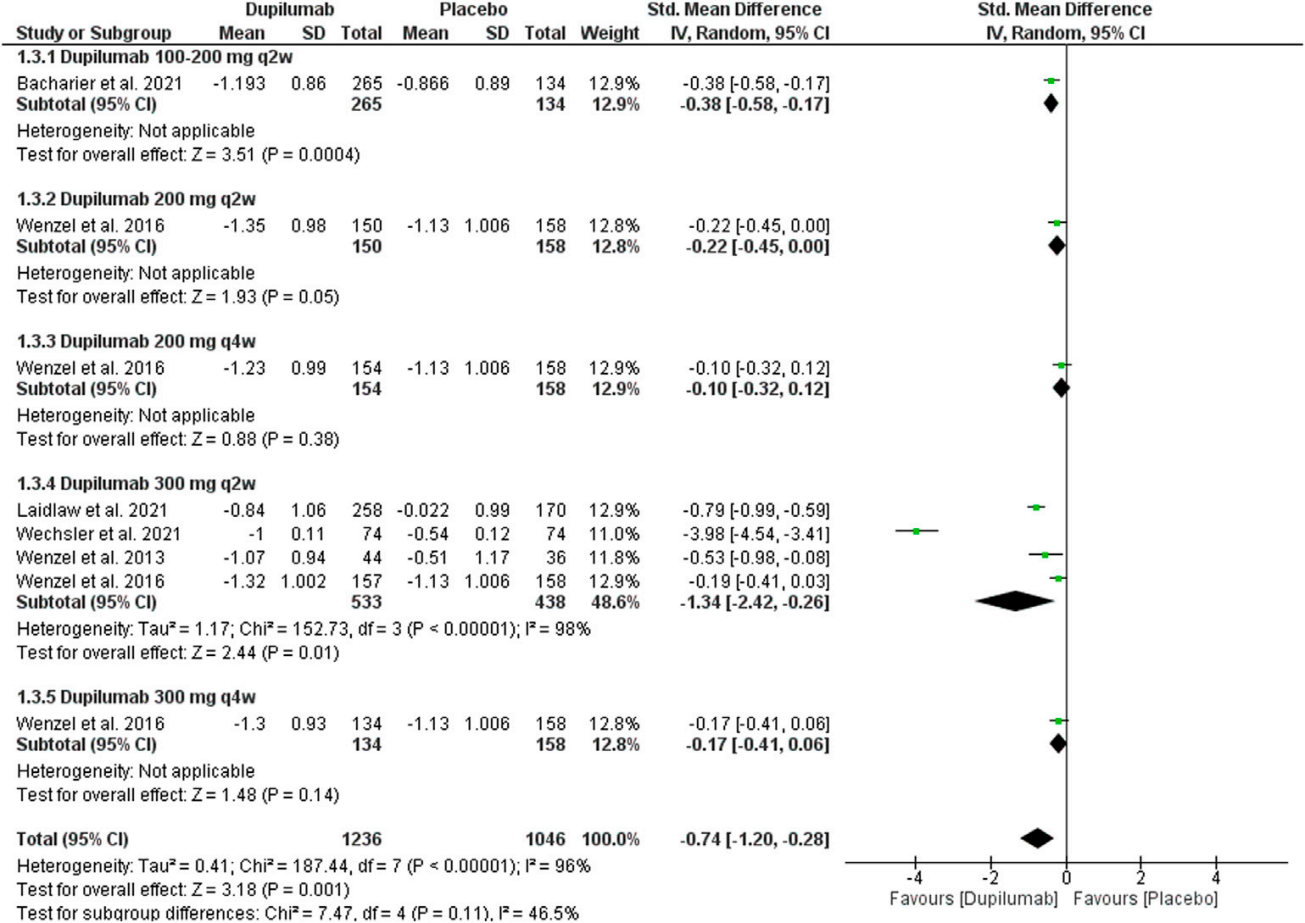
Results of the ACQ change at the 12th week.

This outcome was reported in five trials (Wenzel et al., 2013; Wenzel et al., 2016; Bacharier et al., 2021; Laidlaw et al., 2021; Wechsler et al., 2021). They revealed a significant reduction in the absolute ACQ score at 12 weeks In the dupilumab group compared with the placebo; MD = −0.74 (95% CI = −1.20, −0.28), *p* = 0.001. The overall analysis revealed heterogeneity *p* < 0.01, I^2^ = 96%. The subgroup analysis revealed no significant difference between all subgroups, *p* = 0.11 (Figure 5).

Regarding the 300 mg q2w regimen reported by four trials (Wenzel et al., 2013; Wenzel et al., 2016; Laidlaw et al., 2021; Wechsler et al., 2021), the dupilumab revealed a significant reduction in the ACQ score compared with the placebo; MD = −1.34 (95% CI = −2.42, −0.26), *p* < 0.01. This subgroup data were heterogeneous; *p* < 0.001, I^2^ = 98%. The sensitivity analysis could not solve this heterogeneity.

##### 3.3.1.4 Change in ACQ at week 24

**FIGURE 6:**
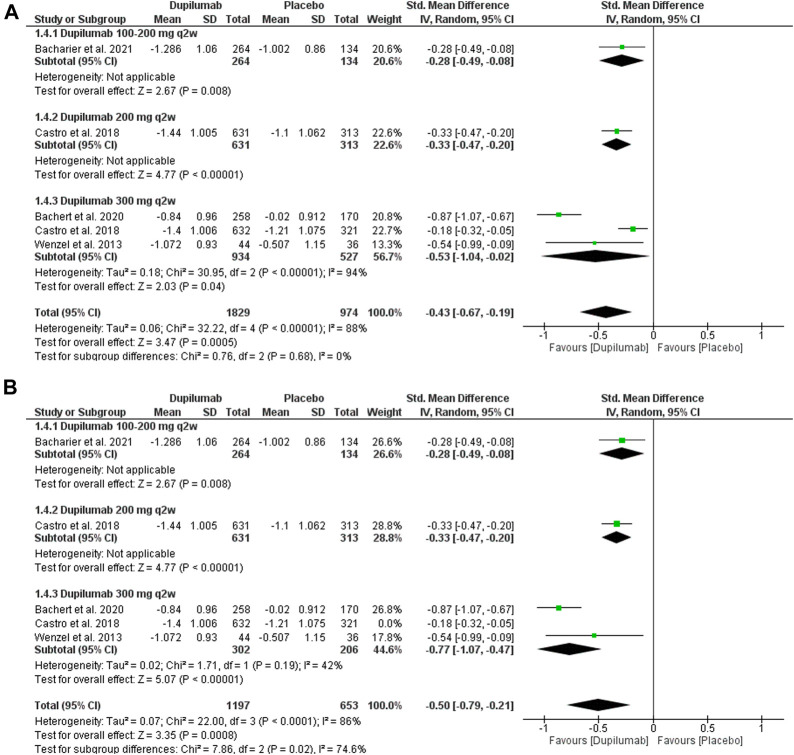
Results of the ACQ change at the 24th week. **(A)** Before sensitivity analysis. **(B)** After sensitivity analysis.

This outcome was reported in four trials (Wenzel et al., 2013; Castro et al., 2018; Bachert et al., 2019; Bacharier et al., 2021). They revealed that dupilumab significantly reduced the absolute ACQ score at 24 weeks Compared with the placebo; MD = −0.43 (95% CI = −0.67, −0.19), *p* = 0.0005. The pooled analysis was heterogeneous; *p* <0.001, I^2^ = 88%. The subgroup analysis revealed no significant difference between subgroups, *p* = 0.68 (Figures 6A,B). As for 300 mg q2w, dupilumab revealed a significant reduction in ACQ at 24 weeks Compared with the placebo; MD = −0.53 (95% CI = −1.04, −0.02), *p* = 0.04. This subgroup pooled data were heterogeneous; *p* < 0.001, I^2^ = 94%. To solve this heterogeneity, we excluded Castro et al. (2018). After sensitivity analysis, there was a significant ACQ score reduction in the dupilumab group compared with the placebo; MD = −0.77 (95% CI = −1.07, −0.47), *p* < 0.001. The subgroup analysis was homogeneous; *p* = 0.19, I^2^ = 42%.

#### 3.3.2 Change in biomarkers of asthma after 12 and 24 weeks

##### 3.3.2.1 Change in FeNO (ppb) at week 12

Six trials (Wenzel et al., 2013; Wenzel et al., 2016; Castro et al., 2018; Rabe et al., 2018; Bacharier et al., 2021; Wechsler et al., 2021) reported this outcome. Dupilumab significantly reduced the FeNO (ppb) compared with the placebo; MD = −17.58 (95% CI = −21.87, −13.29), *p* < 0.001. The pooled analysis was heterogeneous *p* = 0.005, I^2^ = 62%. The subgroup analysis revealed no significant difference between those regimens regarding the dupilumab efficacy, *p* = 0.84 (Supplementary Figure S1).

Regarding the 200 mg q2w regimens, the pooled two trials (Wenzel et al., 2016; Castro et al., 2018) revealed no significant reduction in the FeNO (ppb) compared with the placebo; MD = −22.25 (95% CI = −44.73, 0.23), *p* = 0.05. The pooled analysis was heterogeneous, *p* = 0.01, I^2^ = 84%.

Five trials (Wenzel et al., 2013; Wenzel et al., 2016; Castro et al., 2018; Rabe et al., 2018; Wechsler et al., 2021) evaluated the 300 mg q2w regimen. Dupilumab significantly reduced the FeNO (ppb) compared with the placebo; MD = −19.56 (95% CI = −27.21, −11.90), *p* < 0.001. The pooled analysis was heterogeneous, *p* = 0.004, I^2^ = 74%. We could not resolve this heterogeneity by sensitivity analysis.

##### 3.3.2.2 Change in FeNO (ppb) at week 24

Four trials reported this outcome (Wenzel et al., 2016; Castro et al., 2018; Rabe et al., 2018; Bacharier et al., 2021). At the week 24, dupilumab significantly reduced the FeNO (ppb) compared with the placebo; MD = −19.50 (95% CI = −24.74, −14.25), *p* < 0.001. The pooled analysis was heterogeneous, *p* = 0.001, I^2^ = 71%. The subgroup analysis revealed no significant difference between them, *p* = 0.88 (Supplementary Figures S2A,B).

Two trials (Wenzel et al., 2016; Castro et al., 2018) reported the 200 mg q2w regimen. They reported a significant reduction in the FeNO (ppb) in the dupilumab group compared with the placebo group; MD = −21.61 (95% CI = −40.37, −2.85), *p* = 0.02. The pooled analysis was heterogeneous, *p* = 0.01, I^2^ = 83%.

In addition, three trials (Wenzel et al., 2016; Castro et al., 2018; Rabe et al., 2018) reported the 300 mg q2w revealing that the dupilumab significantly reduced the FeNO compared with the placebo; MD = −21.18 (95% CI = −33.97, −8.38], *p* = 0.001. The pooled analysis was heterogeneous, *p* = 0.0009, I^2^ = 86%. We solved this heterogeneity by exclusion of Wenzel et al. (2016); *p* = 0.21, I^2^ = 36%. The pooled analysis of subgroup remained significant; MD = −13.34 (95% CI = −18.67, −8.00), *p* < 0.001.

##### 3.3.2.3 Change in blood eosinophils (cells/mm^3^) at week 12

This outcome was reported in four trials (Wenzel et al., 2013; Castro et al., 2018; Bacharier et al., 2021; Wechsler et al., 2021). The placebo group showed significantly lower serum eosinophil levels than the dupilumab group, which showed an increase in their levels; MD = 133.05 (95% CI = 97.46, 168.64), *p* < 0.001. The pooled analysis was homogeneous; *p* = 0.41, I^2^ = 0%. The subgroup analysis showed no significant difference between each group; *p* = 0.51, I^2^ = 0% (Supplementary Figure S3).

In three trials (Wenzel et al., 2013; Castro et al., 2018; Wechsler et al., 2021), the dupilumab 300 mg q2w showed a significant increase in eosinophil levels in the dupilumab group; MD = 168.27, (95% CI = 76.12, 260.41), *p* = 0.0003. The pooled data were homogeneous; *p* = 0.23, I^2^ = 33%.

##### 3.3.2.4 Change in blood eosinophils (cells/mm^3^) at week 24

This outcome was reported in two trials (Castro et al., 2018; Bacharier et al., 2021). Similarly, in the 24th week, the changes in the placebo group were significantly lower than those in the dupilumab group; MD = 94.66 (95% CI = 54.92, 134.40), *p* < 0.001. The pooled analysis was homogeneous; *p* = 0.87, I^2^ = 0%. The subgroups of different dupilumab regimens did not show a significant difference; *p* = 0.87 (Supplementary Figure S4).

##### 3.3.2.5 Change in IgE (IU/ml) at week 12

This outcome was reported in three trials (Wenzel et al., 2013; Castro et al., 2018; Wechsler et al., 2021). The dupilumab significantly reduced the IgE levels compared with the placebo; MD = −149.27 (95% CI = −176.39, −122.16), *p* < 0.001. The pooled data were homogeneous; *p* = 0.34, I^2^ = 11%. The subgroups did not show a significant difference between both regimens; *p* = 0.18 (Supplementary Figure S5).

##### 3.3.2.6 Change in IgE (IU/ml) at week 24

This outcome was reported by three trials (Castro et al., 2018; Bachert et al., 2019; Bacharier et al., 2021). Dupilumab significantly reduced the IgE levels at the 24th week compared with the placebo; MD = −210.28, (95% CI = −365.02, −55.55), *p* = 0.008. The pooled analysis was heterogeneous, *p* < 0.001, I^2^ = 98%. The subgroup analysis revealed no significant difference between dupilumab regimens, *p* = 0.55 (Supplementary Figure S6).

#### 3.3.3 Safety profile of dupilumab

The adverse events of dupilumab were reported by most of the included trials. Compared to the placebo, dupilumab revealed a significantly higher incidence of upper respiratory tract infections (URTI), injection-site reaction, and eosinophilia, *p* < 0.05 (Table 3; Supplementary Figures S7, S14).

**TABLE 3:** Details of the adverse events results of the included studies.

Outcome	Number of studies	Significance			Heterogeneity	
		RR	95% CI	*p*-value	*p*-value	I2 (%)
Any adverse events	5	0.98	[0.95, 1.02]	0.44	0.75	0
Any treatment-emergent adverse events	3	1.07	[0.97, 1.18]	0.15	0.53	0
Any adverse events leading to permanent discontinuation	5	1.01	[0.68, 1.49]	0.98	0.49	0
Any treatment-emergent adverse events leading to permanent discontinuation	2	1.29	[0.67, 2.46]	0.45	0.81	0
Serious adverse events	4	1.01	[0.76, 1.35]	0.93	0.63	0
Serious treatment-emergent adverse events	2	1.25	[0.72, 2.17]	0.42	0.89	0
Any adverse events leading to death	4	1.04	[0.28, 3.81]	0.96	0.47	0
Any treatment-emergent adverse events leading to death	2	1.3	[0.06, 26.92]	0.87	-	
Upper respiratory tract infection	6	0.82	[0.68, 0.99]	**0.03**	0.91	0
Viral upper respiratory tract infection	5	0.88	[0.59, 1.31]	0.52	0.16	39
Influenza	3	0.92	[0.46, 1.84]	0.81	0.11	55
Nasopharyngitis	5	0.94	[0.73, 1.22]	0.66	0.51	0
Sinusitis	4	0.82	[0.47, 1.45]	0.5	0.3	19
Bronchitis	4	0.81	[0.66, 1.00]	0.05	0.71	0
Injection-site reaction	7	1.73	[1.37, 2.19]	**0.0001**	0.21	28
Eosinophilia	2	10.73	[2.59, 44.43]	**0.001**	0.67	0
Headache	6	0.89	[0.71, 1.11]	0.3	0.72	0
Allergic rhinitis	3	0.68	[0.35, 1.33]	0.26	0.12	53
Cough	2	0.57	[0.17, 1.96]	0.37	0.22	35
Urinary tract infection	2	0.66	[0.42, 1.05]	0.08	0.35	0
Back pain	2	1.25	[0.78, 1.99]	0.35	0.43	0
Erythema	2	1.1	[0.70, 1.72]	0.68	0.35	0

RR; risk ratio, CI; confidence interval.

Bold values mean the results show statistical significance.

On the other hand, there was no significant difference between dupilumab and placebo groups (*p* ≥ 0.05), regarding the following outcomes; any adverse events, any treatment-emergent adverse events, any adverse events leading to permanent discontinuation, any treatment-emergent adverse events leading to permanent discontinuation, serious adverse events, serious treatment-emergent adverse events, any adverse events leading to death, any treatment-emergent adverse events leading to death, viral upper respiratory tract infection, influenza, bronchitis, nasopharyngitis, sinusitis, headache, allergic rhinitis, cough, urinary tract infection, back pain, and erythema.

### 3.4 Qualitative synthesis

Four trials (Castro et al., 2020; Corren et al., 2020; Tohda et al., 2020; Corren et al., 2021) were included in our qualitative synthesis. In their 2020 trial, Corren et al. (2020) assessed the efficacy of the dupilumab during a treatment period of 52 weeks in 1,902 patients (allergic and eosinophilic asthma). They found dupilumab reduced asthma exacerbation and the inflammatory biomarkers and FEV1 improvement in both types of asthma. Moreover, in a 2021 trial by Corren et al. (2021), they assessed the same outcomes in patients with more than one, two, or three exacerbations in the year before the trial. In addition, they classified patients in different subgroups according to the baseline blood eosinophils, FeNO, and inhaled corticosteroid doses. Corren et al. (2021) in a 2021 *post hoc* analysis reported similar results to their previous 2020 trial. Another *post hoc* analysis by Castro et al. (2020) investigated how dupilumab affects lung function in total participants and according to inflammatory biomarker levels. They concluded that dupilumab enhances lung function results, especially in patients with increased type-2 inflammatory biomarkers. Tohda et al. (2020), in their 2020 trial, evaluated the efficacy of dupilumab in the Japanese subpopulation of the QUEST trial (114). They found dupilumab reduced asthma exacerbation and the inflammatory and improved FEV1 in the Japanese population of QUEST, indicating the significance of dupilumab in different ethnic groups.

## 4 Discussion

Our pooled data of 13 RCTs found that dupilumab significantly improved the FEV1 at the 12th and 24th weeks. In addition, it reduced FeNO levels, IgE levels, and ACQ scores of asthmatic patients at the 12th and 24th weeks. However, it was associated with an increase in blood eosinophils at the 12th and 24th weeks. Dupilumab was generally a safe agent for asthmatic patients. It showed no significant difference compared with the placebo regarding all adverse effects, except for upper respiratory tract infection, injection-site reaction, and eosinophilia, which had a significantly higher incidence in the dupilumab group. Furthermore, those findings seem to be dose-independent as there was no significant difference between different subgroups.

Those results were consistent with most of the results of these trials (Wenzel et al., 2013; Wenzel et al., 2016; Castro et al., 2018; Rabe et al., 2018; Weinstein et al., 2018; Bachert et al., 2019; Castro et al., 2020; Corren et al., 2020; Tohda et al., 2020; Bacharier et al., 2021; Corren et al., 2021; Laidlaw et al., 2021; Wechsler et al., 2021) and with previous meta-analyses (Edris et al., 2019; Xiong et al., 2019; Zayed et al., 2019). However, Weinstein et al. (2018) trial reported that the 200 mg q2w dupilumab regimen was associated with a statistically insignificant improvement in FEV1 compared with the placebo in patients with perennial allergic rhinitis (PAR). But this was different in non-PAR patients, whereas dupilumab 200 mg/2 weeks regimen significantly increased FEV1 by 0.15 L compared to the placebo. In contrast, the 300 mg q2w dupilumab regimen showed no significant difference regarding FEV1 compared with the placebo. This indicates the importance of classifying asthmatic patients according to their medical conditions or comorbidities and the importance of the choice of treatment regimens. Moreover, similar results were found regarding the annualized rate of severe exacerbations.

The effect of dupilumab starts early after the beginning of the treatment course and is maintained to the end of the follow-up periods of different RCTs up to 52 weeks, as reported by most of our included studies. In addition, it is reported that dupilumab reduced the annualized rate of severe asthma exacerbations by 47%, especially when added to inhaled corticosteroids and other controllers compared with the placebo (Castro et al., 2018).

Asthmatic children require special attention as uncontrolled asthma affects pulmonary functions and limits the airflow, leading to COPD in adulthood (Tagiyeva et al., 2016; McGeachie, 2017). However, the protective role of dupilumab against COPD development or restoring normal pulmonary growth and function is still unclear. Similarly, the role of the different treatment doses and duration is still a query. Although there was no significant difference between different regimens of dupilumab in our trial, we think that the actual effect may be detected in the long-term course of treatment beyond our follow-up periods.

Furthermore, Bacharier et al. (2021) reported that 78% of children using dupilumab as an add-on therapy experienced an exacerbation-free period during the 52 weeks of treatment, compared with 60% of children in the placebo group. Those patients required less use of systemic corticosteroids. This is a critical indicator of efficacy and safety, especially among pediatric asthmatic patients, as they avoided the long-term use of corticosteroids with their subsequent complications.

IL-13 promotes the activity of NO-synthase with increased NO levels. This indicates NO’s role as a biomarker of asthmatic activity and could be correlated to the levels of IL in the airway mucosa (Chibana et al., 2008; Barranco et al., 2017). Our results were consistent with these mechanisms. We observed that dupilumab significantly reduced the local and systemic inflammatory biomarkers such as FeNO and IgE. This confirms the role of dupilumab in the inflammatory process signaling and activity. In addition, those biomarkers may be used as a screening test for the response to dupilumab and other agents targeting the type-2 inflammatory pathway.

On the other hand, our pooled analysis revealed a relatively higher serum eosinophilic count in the dupilumab group compared with the placebo. This increase seems to be transient at the beginning of dupilumab treatment in adults (Castro et al., 2018) and children (Bacharier et al., 2021). During the inflammatory process, the IL-4 and IL-13 produce eotaxin and vascular cell adhesion molecule, which stimulates eosinophils’ migration to targeted tissues. Dupilumab blocks this sequence of events retaining eosinophils in the circulation (Barthel et al., 2008; Tozawa et al., 2011). Moreover, Rabe et al. (2018) explained the increase in blood eosinophil levels due to different corticosteroid dosages between both study arms as glucocorticoids reduce the levels of blood eosinophils. In the dupilumab group, the dose of the glucocorticoids was reduced compared with that in the placebo group. This seems to be responsible for eosinophilia. In addition, the elevation of blood eosinophil levels was not associated with clinical consequences; it was only a laboratory finding, as reported by Castro et al. (2018). Nevertheless, we think dupilumab is still effective in treating asthma, as it significantly reduces the key inflammatory mediators. However, it causes eosinophilia but of no significant role in the efficacy of the dupilumab against inflammation.

**FIGURE 7:**
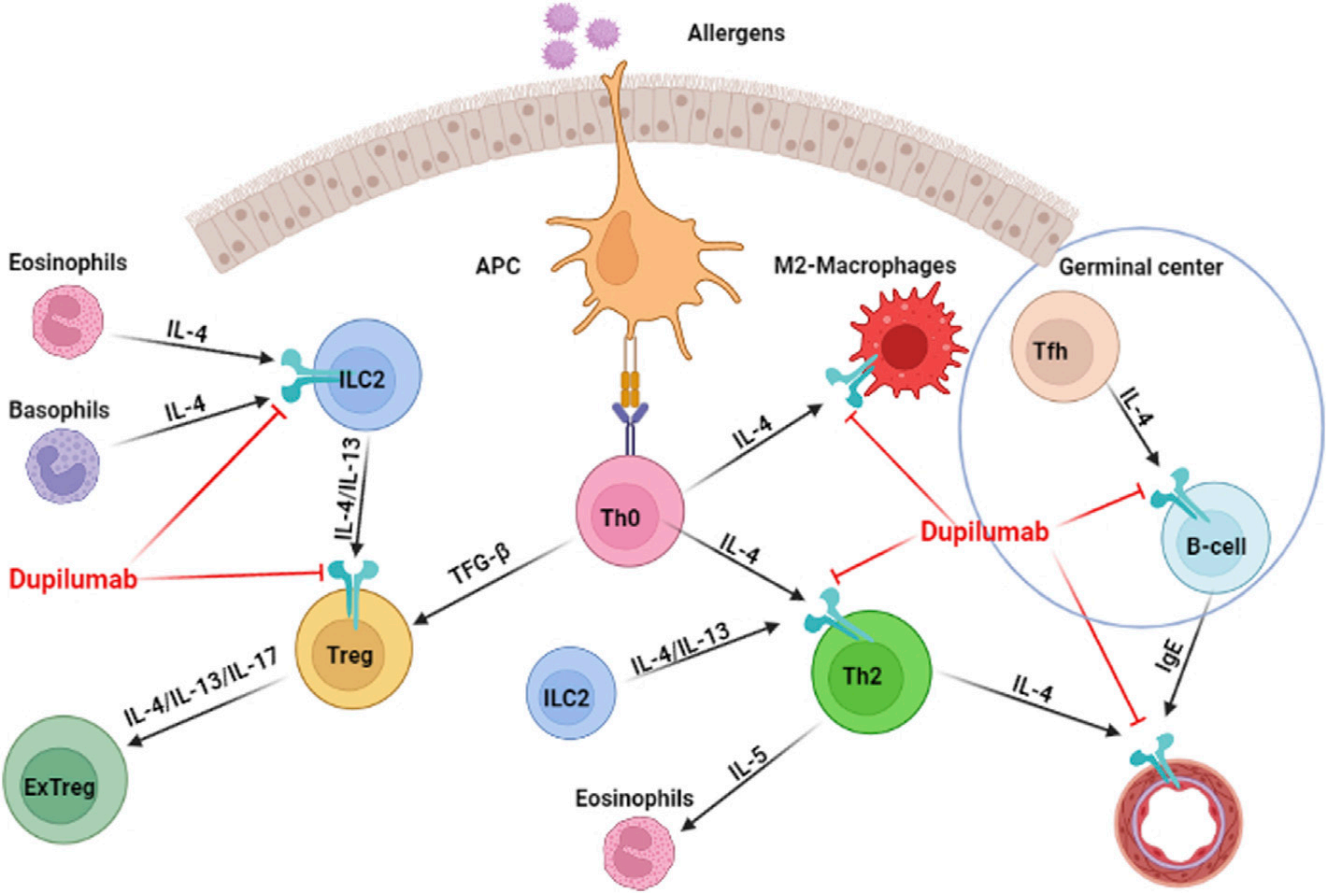
Mechanism of action of dupilumab through different sites.

The literature lacks the exact mechanism of action of dupilumab either *in vivo* or *in vitro* studies (Harb and Chatila, 2020). Dupilumab is a human monoclonal antibody directed against the alpha subunit of the interleukin-4 receptor and inhibits the signaling of IL-4 and IL-13 (Le Floc’h et al., 2020). Dupilumab acts on the alpha subunit of IL-4 receptor and prevents the binding of the IL-4 to type 1 receptor. In addition, it may inhibit the protein assembly of the type-2 receptor complex. This process may be explained by the inhibition of binding of IL-13 to IL-13 receptor, which is needed for the mobilization of the IL-4 alpha receptor. Moreover, the binding of IL-4 and 13 to their targeted receptors conducts a series of events leading to the recruitment of the other receptors subunits (Harb and Chatila, 2020). Dupilumab has different sites of action, which are fundamental for the Th2 inflammatory process of various diseases. Apart from inflammatory cells, it can also act on endothelium, reducing the cellular recruitment and vascular permeability for those cells (Harb and Chatila, 2020) (Figure 7).

Viral infections have been shown to exacerbate asthma symptoms. Few data exist on COVID-19 immunological responses in biologic-treated asthmatics, and using biological agents during the COVID-19 pandemic is still debatable. A multicenter study by Eger et al. (2020) revealed that biologic-treated asthmatics were highly vulnerable to COVID-19 infection and had a higher severity than the general population. In contrast, other studies (Klimek et al., 2020; Patruno et al., 2020; Bhalla et al., 2021; Grieco et al., 2021; Tanabe et al., 2021; Ungar et al., 2022a; Ungar et al., 2022b) reported that dupilumab is a safe agent to use during the COVID-19 pandemic and may reduce the severity of COVID-19 symptoms. However, the role of biological agents on the COVID-19 response is still unclear, so each patient should be carefully assessed, and the patient should be involved in considering the therapy’s benefits and hazards.

Regarding the other adverse events, all the included studies reported similar incidence of adverse events in both dupilumab and placebo regarding most adverse events. The injection-site reaction was increased with dupilumab treatment in addition to upper respiratory tract infection. This points to the importance of reaching an explanation of those inflammatory conditions in a drug that is thought to reduce inflammation. This may be due to different mechanisms related to the treatment regimen, duration, or the associated add-ons; however, it is not reported. Reaching an explanation of such adverse events is essential to avoid them and to reach more efficient results while manufacturing those agents.

The heterogeneity of some outcomes is the main limitation of this meta-analysis. This heterogeneity might be due to some of the included studies including asthmatic patients associated with other type-2 inflammatory diseases. The dupilumab regimens were not reported in multiple studies, so we could not have a definite conclusion about all regimens. Also, some of the included studies were *post hoc* analyses; thus, we could not pool these data when the results were reported in the original one. In addition, we could not conduct an age-dependent analysis of the efficacy of the dupilumab on different outcomes to determine the best age group to benefit from the targeted medication. There was no data beyond 52 weeks of treatment, and we could not determine the least period for the most effective results. Also, the pediatric population needs special care to detect the least effective dose to avoid the toxic doses. Other trials comparing dupilumab with the placebo and other drugs are needed. We suggest further RCTs that assess the safety and efficacy of dupilumab according to the biomarker level of type-2 inflammation, types of asthma, and age groups. Furthermore, a network meta-analysis to compare dupilumab with other standard treatments is recommended to show the best option for asthmatic patients.

## 5 Conclusion

Dupilumab improves pulmonary function and reduces local and systemic inflammatory markers with minimal adverse events in patients with moderate to severe asthma. Those effects seem to be dose-independent as there was no significant difference between different regimen subgroups.

## Data Availability

The original contributions presented in the study are included in the article/Supplementary Material; further inquiries can be directed to the corresponding author.

## References

[B1] Arrieta M-C. Stiemsma L. T. Dimitriu P. A. Thorson L. Russell S. Yurist-Doutsch S. (2015). Early infancy microbial and metabolic alterations affect risk of childhood asthma. Sci. Transl. Med. 7 (307), 307ra152. 10.1126/scitranslmed.aab2271 26424567

[B2] Bacharier L. B. Maspero J. F. Katelaris C. H. Fiocchi A. G. Gagnon R. de Mir I. (2021). Dupilumab in children with uncontrolled moderate-to-severe asthma. N. Engl. J. Med. 385 (24), 2230–2240. 10.1056/NEJMoa2106567 34879449

[B3] Bachert C. Han J. K. Desrosiers M. Hellings P. W. Amin N. Lee S. E. (2019). Efficacy and safety of dupilumab in patients with severe chronic rhinosinusitis with nasal polyps (LIBERTY NP SINUS-24 and LIBERTY NP SINUS-52): Results from two multicentre, randomised, double-blind, placebo-controlled, parallel-group phase 3 trials. Lancet 394 (10209), 1638–1650. 10.1016/S0140-6736(19)31881-1 31543428

[B4] Barranco P. Phillips-Angles E. Dominguez-Ortega J. Quirce S. (2017). Dupilumab in the management of moderate-to-severe asthma: The data so far. Ther. Clin. Risk Manag. 13, 1139–1149. 10.2147/TCRM.S125964 28979129 PMC5589101

[B5] Barthel S. R. Johansson M. W. McNamee D. M. Mosher D. F. (2008). Roles of integrin activation in eosinophil function and the eosinophilic inflammation of asthma. J. Leukoc. Biol. 83 (1), 1–12. 10.1189/jlb.0607344 17906117 PMC2859217

[B6] Bellin M. H. Osteen P. Kub J. Bollinger M. E. Tsoukleris M. Chaikind L. (2015). Stress and quality of life in urban caregivers of children with poorly controlled asthma: A longitudinal analysis. J. Pediatr. Health Care. 29 (6), 536–546. 10.1016/j.pedhc.2015.04.018 26036621 PMC4624025

[B7] Bhalla A. Mukherjee M. Radford K. Nazy I. Kjarsgaard M. Bowdish D. M. E. (2021). Dupilumab, severe asthma airway responses, and SARS-CoV-2 serology. Allergy 76 (3), 957–958. 10.1111/all.14534 32767400 PMC7436521

[B8] Castro M. Corren J. Pavord I. D. Maspero J. Wenzel S. Rabe K. F. (2018). Dupilumab efficacy and safety in moderate-to-severe uncontrolled asthma. N. Engl. J. Med. 378 (26), 2486–2496. 10.1056/NEJMoa1804092 29782217

[B9] Castro M. Rabe K. F. Corren J. Pavord I. D. Katelaris C. H. Tohda Y. (2020). Dupilumab improves lung function in patients with uncontrolled, moderate-to-severe asthma. ERJ Open Res. 6 (1), 00204–02019. 10.1183/23120541.00204-2019 PMC6983496 32010719

[B10] Chauhan B. F. Ducharme F. M. (2014). Addition to inhaled corticosteroids of long acting beta 2 agonists versus anti leukotrienes for chronic asthma. Cochrane Database Syst. Rev. (1), CD003137. 10.1002/14651858.CD003137.pub5 24459050 PMC10514761

[B11] Chen Z. Salam M. T. Alderete T. L. Habre R. Bastain T. M. Berhane K. (2017). Effects of childhood asthma on the development of obesity among school-aged children. Am. J. Respir. Crit. Care Med. 195 (9), 1181–1188. 10.1164/rccm.201608-1691OC 28103443 PMC5439015

[B12] Chibana K. Trudeau J. B. Mustovich A. T. Hu H. Zhao J. Balzar S. (2008). IL-13 induced increases in nitrite levels are primarily driven by increases in inducible nitric oxide synthase as compared with effects on arginases in human primary bronchial epithelial cells. Clin. Exp. Allergy 38 (6), 936–946. 10.1111/j.1365-2222.2008.02969.x 18384429 PMC11934259

[B13] Corren J. Castro M. O'Riordan T. Hanania N. A. Pavord I. D. Quirce S. (2020). Dupilumab efficacy in patients with uncontrolled, moderate-to-severe allergic asthma. J. Allergy Clin. Immunol. Pract. 8 (2), 516–526. 10.1016/j.jaip.2019.08.050 31521831

[B14] Corren J. Katelaris C. H. Castro M. Maspero J. F. Ford L. B. Halpin D. M. G. (2021). Effect of exacerbation history on clinical response to dupilumab in moderate-to-severe uncontrolled asthma. Eur. Respir. J. 58 (4), 2004498. 10.1183/13993003.04498-2020 34266940 PMC8551561

[B15] Edris A. De Feyter S. Maes T. Joos G. Lahousse L. (2019). Monoclonal antibodies in type 2 asthma: A systematic review and network meta-analysis. Respir. Res. 20 (1), 179. 10.1186/s12931-019-1138-3 31395084 PMC6688359

[B16] Eger K. Hashimoto S. Braunstahl G. J. Brinke A. T. Patberg K. W. Beukert A. (2020). Poor outcome of SARS-CoV-2 infection in patients with severe asthma on biologic therapy. Respir. Med. 177, 106287. 10.1016/j.rmed.2020.106287 33388603 PMC7833566

[B17] Fahy J. V. (2015). Type 2 inflammation in asthma—Present in most, absent in many. Nat. Rev. Immunol. 15 (1), 57–65. 10.1038/nri3786 25534623 PMC4390063

[B18] Fleming L. Murray C. Bansal A. T. Hashimoto S. Bisgaard H. Bush A. (2015). The burden of severe asthma in childhood and adolescence: Results from the paediatric U-biopred cohorts. Eur. Respir. J. 46 (5), 1322–1333. 10.1183/13993003.00780-2015 26405287

[B19] Grieco T. Chello C. Sernicola A. Muharremi R. Michelini S. Paolino G. (2021). Impact of COVID-19 on patients with atopic dermatitis. Clin. Dermatol. 39 (6), 1083–1087. 10.1016/j.clindermatol.2021.07.008 34920828 PMC8285243

[B20] Harb H. Chatila T. A. (2020). Mechanisms of dupilumab. Clin. Exp. Allergy 50 (1), 5–14. 10.1111/cea.13491 31505066 PMC6930967

[B21] Higgins J. P. Altman D. G. Gøtzsche P. C. Jüni P. Moher D. Oxman A. D. (2011). The Cochrane Collaboration's tool for assessing risk of bias in randomised trials. Bmj 343, d5928. 10.1136/bmj.d5928 22008217 PMC3196245

[B22] Higgins J. P. T. (2019). Cochrane handbook for systematic reviews of interventions. Second edition. Hoboken, NJ: Wiley-Blackwell.

[B23] Jl Murray C. (2020). Global burden of 369 diseases and injuries in 204 countries and territories, 1990–2019: A systematic analysis for the global burden of disease study 2019. Lancet 396 (10258), 1204–1222. 10.1016/S0140-6736(20)30925-9 33069326 PMC7567026

[B24] Klimek L. Pfaar O. Worm M. Eiwegger T. Hagemann J. Ollert M. (2020). Use of biologicals in allergic and type-2 inflammatory diseases during the current COVID-19 pandemic: Position paper of Ärzteverband Deutscher Allergologen (AeDA)(A), Deutsche Gesellschaft für Allergologie und Klinische Immunologie (DGAKI)(B), Gesellschaft für Pädiatrische Allergologie und Umweltmedizin (GPA)(C), Österreichische Gesellschaft für Allergologie und Immunologie (ÖGAI)(D), Luxemburgische Gesellschaft für Allergologie und Immunologie (LGAI)(E), Österreichische Gesellschaft für Pneumologie (ÖGP)(F) in co-operation with the German, Austrian, and Swiss ARIA groups(G), and the European Academy of Allergy and Clinical Immunology (EAACI)(H). Allergol. Sel. 4, 53–68. 10.5414/ALX02166E PMC7480069 32915172

[B25] Laidlaw T. M. Bachert C. Amin N. Desrosiers M. Hellings P. W. Mullol J. (2021). Dupilumab improves upper and lower airway disease control in chronic rhinosinusitis with nasal polyps and asthma. Ann. Allergy Asthma Immunol. 126 (5), 584–592.e1. 10.1016/j.anai.2021.01.012 33465455

[B26] Le Floc’h A. Allinne J. Nagashima K. Scott G. Birchard D. Asrat S. (2020). Dual blockade of IL‐4 and IL‐13 with dupilumab, an IL‐4Rα antibody, is required to broadly inhibit type 2 inflammation. Allergy 75 (5), 1188–1204. 10.1111/all.14151 31838750 PMC7317958

[B27] McGeachie M. J. (2017). Childhood asthma is a risk factor for the development of chronic obstructive pulmonary disease. Curr. Opin. Allergy Clin. Immunol. 17 (2), 104–109. 10.1097/ACI.0000000000000348 28118239 PMC5577926

[B28] Newman S. P. (2004). Spacer devices for metered dose inhalers. Clin. Pharmacokinet. 43 (6), 349–360. 10.2165/00003088-200443060-00001 15086274

[B29] Page M. J. McKenzie J. E. Bossuyt P. M. Boutron I. Hoffmann T. C. Mulrow C. D. (2021). The PRISMA 2020 statement: An updated guideline for reporting systematic reviews. Bmj 372, n71. 10.1136/bmj.n71 33782057 PMC8005924

[B30] Patruno C. Stingeni L. Fabbrocini G. Hansel K. Napolitano M. (2020). Dupilumab and COVID-19: What should we expect? Dermatol. Ther. 33 (4), e13502. 10.1111/dth.13502 32362061 PMC7267436

[B31] Rabe K. F. Nair P. Brusselle G. Maspero J. F. Castro M. Sher L. (2018). Efficacy and safety of dupilumab in glucocorticoid-dependent severe asthma. N. Engl. J. Med. 378 (26), 2475–2485. 10.1056/NEJMoa1804093 29782224

[B32] Reddel H. K. Bacharier L. B. Bateman E. D. Brightling C. E. Brusselle G. G. Buhl R. (2022). Global initiative for asthma strategy 2021: Executive summary and rationale for key changes. Eur. Respir. J. 59 (1), 2102730. 10.1183/13993003.02730-2021 34667060 PMC8719459

[B33] Robinson D. S. Hamid Q. Ying S. Tsicopoulos A. Barkans J. Bentley A. M. (1992). Predominant TH2-like bronchoalveolar T-lymphocyte population in atopic asthma. N. Engl. J. Med. 326 (5), 298–304. 10.1056/NEJM199201303260504 1530827

[B34] Steinke J. W. B. L. Borish L. (2001). Th2 cytokines and asthma. Interleukin-4: Its role in the pathogenesis of asthma, and targeting it for asthma treatment with interleukin-4 receptor antagonists. Respir. Res. 2 (2), 66–70. 10.1186/rr40 11686867 PMC59570

[B35] Tagiyeva N. D. G. Fielding S. Turner S. Douglas G. (2016). Outcomes of childhood asthma and wheezy bronchitis. A 50-year cohort study. Am. J. Respir. Crit. Care Med. 193, 23–30. 10.1164/rccm.201505-0870OC 26351837 PMC4731615

[B36] Tanabe N. Matsumoto H. Hamada S. Ito I. Hirai T. (2021). Dupilumab maintenance therapy in an asthmatic patient with coronavirus disease 2019 pneumonia. Allergol. Int. 70 (2), 274–276. 10.1016/j.alit.2020.10.005 33208274 PMC7648507

[B37] Tohda Y. Nakamura Y. Fujisawa T. Ebisawa M. Arima K. Miyata M. (2020). Dupilumab efficacy and safety in Japanese patients with uncontrolled, moderate-to-severe asthma in the phase 3 LIBERTY ASTHMA QUEST study. Allergol. Int. 69 (4), 578–587. 10.1016/j.alit.2020.04.002 32444306

[B38] Tozawa H. Kanki Y. Suehiro J. Tsutsumi S. Kohro T. Wada Y. (2011). Genome-wide approaches reveal functional interleukin-4-inducible STAT6 binding to the vascular cell adhesion molecule 1 promoter. Mol. Cell. Biol. 31 (11), 2196–2209. 10.1128/MCB.01430-10 21464207 PMC3133239

[B39] Ungar B. Lavin L. Golant A. K. Gontzes A. David E. Estrada Y. D. (2022). The impact of dupilumab treatment on severe acute respiratory syndrome coronavirus 2-coronavirus disease 2019 antibody responses in patients with atopic dermatitis. Ann. Allergy Asthma Immunol. 128 (6), 734–736. 10.1016/j.anai.2022.03.019 35346880 PMC8956356

[B40] Ungar B. Glickman J. W. Golant A. K. Dubin C. Marushchak O. Gontzes A. (2022). COVID-19 symptoms are attenuated in moderate-to-severe atopic dermatitis patients treated with dupilumab. J. Allergy Clin. Immunol. Pract. 10 (1), 134–142. 10.1016/j.jaip.2021.10.050 34737108 PMC8558098

[B41] Wechsler M. E. Ruddy M. K. Pavord I. D. Israel E. Rabe K. F. Ford L. B. (2021). Efficacy and safety of itepekimab in patients with moderate-to-severe asthma. N. Engl. J. Med. 385 (18), 1656–1668. 10.1056/NEJMoa2024257 34706171

[B42] Weinstein S. F. Katial R. Jayawardena S. Pirozzi G. Staudinger H. Eckert L. (2018). Efficacy and safety of dupilumab in perennial allergic rhinitis and comorbid asthma. J. Allergy Clin. Immunol. 142 (1), 171–177.e1. 10.1016/j.jaci.2017.11.051 29355679

[B43] Wenzel S. Ford L. Pearlman D. Spector S. Sher L. Skobieranda F. (2013). Dupilumab in persistent asthma with elevated eosinophil levels. N. Engl. J. Med. 368 (26), 2455–2466. 10.1056/NEJMoa1304048 23688323

[B44] Wenzel S. Castro M. Corren J. Maspero J. Wang L. Zhang B. (2016). Dupilumab efficacy and safety in adults with uncontrolled persistent asthma despite use of medium-to-high-dose inhaled corticosteroids plus a long-acting β2 agonist: A randomised double-blind placebo-controlled pivotal phase 2b dose-ranging trial. Lancet 388 (10039), 31–44. 10.1016/S0140-6736(16)30307-5 27130691

[B45] Wu T. D. Brigham E. P. McCormack M. C. (2019). Asthma in the primary care setting. Med. Clin. North Am. 103 (3), 435–452. 10.1016/j.mcna.2018.12.004 30955512 PMC6776421

[B46] Xiong X. F. Zhu M. Wu H. X. Fan L. L. Cheng D. Y. (2019). Efficacy and safety of dupilumab for the treatment of uncontrolled asthma: A meta-analysis of randomized clinical trials. Respir. Res. 20 (1), 108. 10.1186/s12931-019-1065-3 31151443 PMC6544936

[B47] Zayed Y. Kheiri B. Banifadel M. Hicks M. Aburahma A. Hamid K. (2019). Dupilumab safety and efficacy in uncontrolled asthma: A systematic review and meta-analysis of randomized clinical trials. J. Asthma 56 (10), 1110–1119. 10.1080/02770903.2018.1520865 30273510

