# Supplementary material for: Dupilumab efficacy and safety in patients with moderate to severe asthma: A systematic review and meta-analysis
Source: Front Pharmacol. 2022 Oct 3;13:992731. doi: 10.3389/fphar.2022.992731 (PMC9574251; doi:10.3389/fphar.2022.992731)

**Supplementary Figure1:** A forest plot of the change in FENO (ppb) at week 12.

| Study or Subgroup | Dupilumab Mean | Dupilumab SD | Dupilumab Total | Placebo Mean | Placebo SD | Placebo Total | Weight | Mean Difference IV, Random, 95% CI |
|---|---|---|---|---|---|---|---|---|
| **1.5.1 Dupilumab 100-200 mg q2w** | | | | | | | | |
| Bacharier et al. 2021 | -16.95 | 24.49 | 247 | -0.15 | 32.57 | 126 | 14.3% | -16.80 [-23.26, -10.34] |
| **Subtotal (95% CI)** | | | **247** | | | **126** | **14.3%** | **-16.80 [-23.26, -10.34]** |
| Heterogeneity: Not applicable | | | | | | | | |
| Test for overall effect: Z = 5.10 (P < 0.00001) | | | | | | | | |
| **1.5.2 Dupilumab 200 mg q2w** | | | | | | | | |
| Castro et al. 2018 | -14.9 | 31.3 | 631 | -2.5 | 21 | 313 | 18.8% | -12.40 [-15.77, -9.03] |
| Wenzel et al. 2016 | -24.02 | 76.4 | 150 | 11.58 | 77 | 131 | 4.5% | -35.60 [-53.58, -17.62] |
| **Subtotal (95% CI)** | | | **781** | | | **444** | **23.2%** | **-22.25 [-44.73, 0.23]** |
| Heterogeneity: Tau² = 225.55; Chi² = 6.18, df = 1 (P = 0.01); I² = 84% | | | | | | | | |
| Test for overall effect: Z = 1.94 (P = 0.05) | | | | | | | | |
| **1.5.3 Dupilumab 200 mg q4w** | | | | | | | | |
| Wenzel et al. 2016 | 2.45 | 76.34 | 114 | 11.58 | 77 | 131 | 4.0% | -9.13 [-28.37, 10.11] |
| **Subtotal (95% CI)** | | | **114** | | | **131** | **4.0%** | **-9.13 [-28.37, 10.11]** |
| Heterogeneity: Not applicable | | | | | | | | |
| Test for overall effect: Z = 0.93 (P = 0.35) | | | | | | | | |
| **1.5.4 Dupilumab 300 mg q2w** | | | | | | | | |
| Castro et al. 2018 | -15.6 | 25.3 | 632 | -3.6 | 29.6 | 321 | 18.2% | -12.00 [-15.79, -8.21] |
| Rape et al. 2018 | -18.7 | 28.24 | 83 | -2.755 | 31.34 | 93 | 11.2% | -15.95 [-24.75, -7.14] |
| Wechsler et al. 2021 | -7.908 | 19.19 | 58 | 6.906 | 26.46 | 49 | 11.1% | -14.81 [-23.72, -5.91] |
| Wenzel et al. 2013 | -17.862 | 26.9 | 40 | 10.363 | 24.2 | 44 | 8.9% | -28.22 [-39.21, -17.24] |
| Wenzel et al. 2016 | -28.04 | 77.28 | 157 | 11.58 | 77 | 131 | 4.5% | -39.62 [-57.51, -21.73] |
| **Subtotal (95% CI)** | | | **970** | | | **638** | **53.9%** | **-19.56 [-27.21, -11.90]** |
| Heterogeneity: Tau² = 51.33; Chi² = 15.29, df = 4 (P = 0.004); I² = 74% | | | | | | | | |
| Test for overall effect: Z = 5.01 (P < 0.00001) | | | | | | | | |
| **1.5.5 Dupilumab 300 mg q4w** | | | | | | | | |
| Wenzel et al. 2016 | -9.97 | 76.23 | 157 | 11.58 | 77 | 131 | 4.6% | -21.55 [-39.33, -3.77] |
| **Subtotal (95% CI)** | | | **157** | | | **131** | **4.6%** | **-21.55 [-39.33, -3.77]** |
| Heterogeneity: Not applicable | | | | | | | | |
| Test for overall effect: Z = 2.38 (P = 0.02) | | | | | | | | |
| **Total (95% CI)** | | | **2269** | | | **1470** | **100.0%** | **-17.58 [-21.87, -13.29]** |
| Heterogeneity: Tau² = 22.54; Chi² = 23.52, df = 9 (P = 0.005); I² = 62% | | | | | | | | |
| Test for overall effect: Z = 8.04 (P < 0.00001) | | | | | | | | |
| Test for subgroup differences: Chi² = 1.40, df = 4 (P = 0.84), I² = 0% | | | | | | | | |

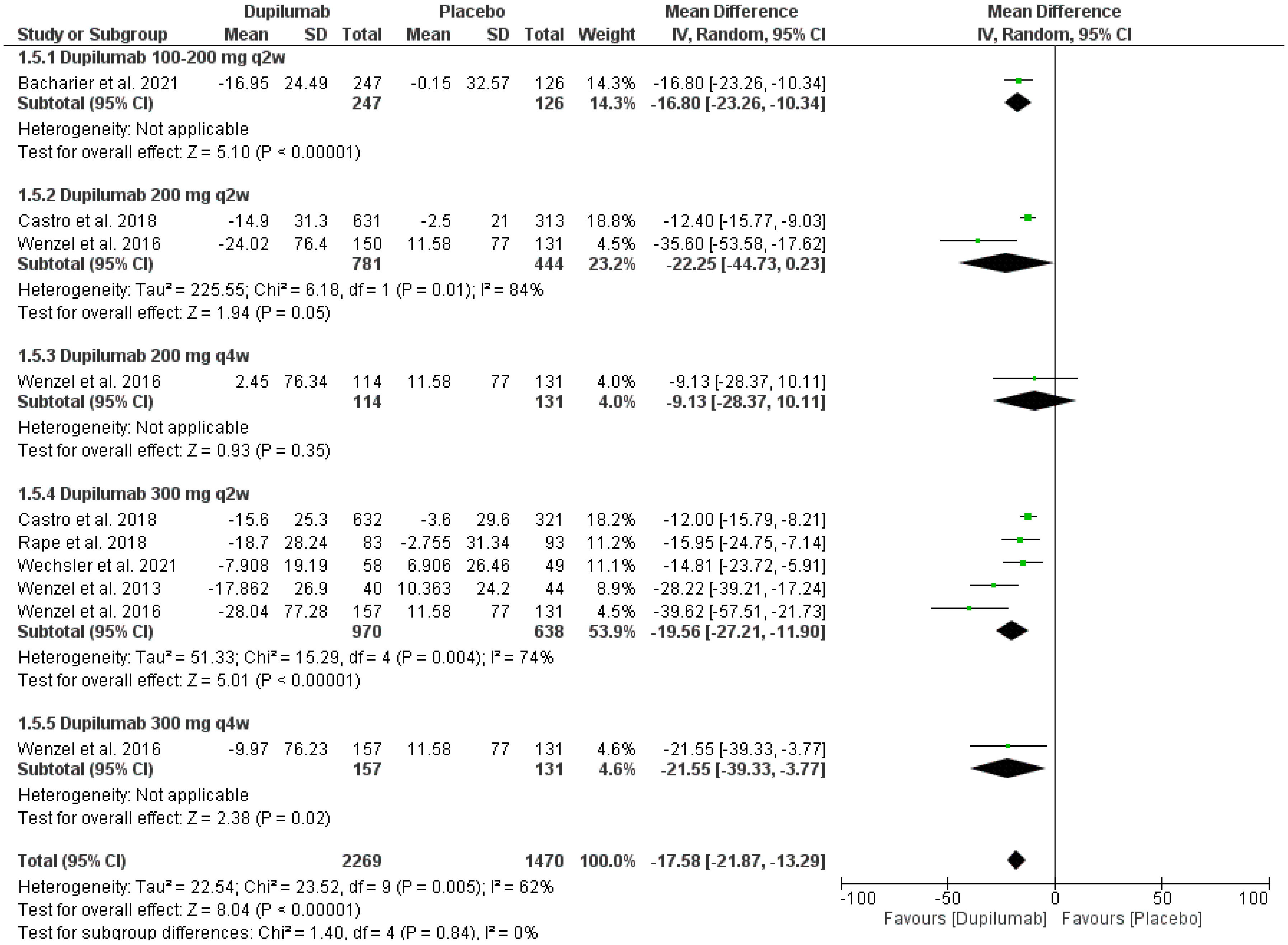

**Supplementary Figures 2A:** A forest plot of the change in FENO (ppb) at week 24.

| Study or Subgroup | Dupilumab Mean | Dupilumab SD | Dupilumab Total | Placebo Mean | Placebo SD | Placebo Total | Weight | Mean Difference IV, Random, 95% CI |
|---|---|---|---|---|---|---|---|---|
| **1.6.1 Dupilumab 100-200 mg q2w** | | | | | | | | |
| Bacharier et al. 2021 | -16.8 | 24.77 | 242 | 2.66 | 37.54 | 123 | 15.4% | -19.46 [-26.79, -12.13] |
| **Subtotal (95% CI)** | | | **242** | | | **123** | **15.4%** | **-19.46 [-26.79, -12.13]** |
| Heterogeneity: Not applicable | | | | | | | | |
| Test for overall effect: Z = 5.20 (P < 0.00001) | | | | | | | | |
| **1.6.2 Dupilumab 200 mg q2w** | | | | | | | | |
| Castro et al. 2018 | -16.2 | 32.6 | 631 | -2.8 | 21.2 | 313 | 20.1% | -13.40 [-16.86, -9.94] |
| Wenzel et al. 2016 | -21.86 | 59.68 | 114 | 10.91 | 59 | 120 | 7.7% | -32.77 [-47.98, -17.56] |
| **Subtotal (95% CI)** | | | **745** | | | **433** | **27.8%** | **-21.61 [-40.37, -2.85]** |
| Heterogeneity: Tau² = 155.91; Chi² = 5.92, df = 1 (P = 0.01); I² = 83% | | | | | | | | |
| Test for overall effect: Z = 2.26 (P = 0.02) | | | | | | | | |
| **1.6.3 Dupilumab 200 mg q4w** | | | | | | | | |
| Wenzel et al. 2016 | -5.47 | 58 | 102 | 10.91 | 59 | 120 | 7.6% | -16.38 [-31.81, -0.95] |
| **Subtotal (95% CI)** | | | **102** | | | **120** | **7.6%** | **-16.38 [-31.81, -0.95]** |
| Heterogeneity: Not applicable | | | | | | | | |
| Test for overall effect: Z = 2.08 (P = 0.04) | | | | | | | | |
| **1.6.4 Dupilumab 300 mg q2w** | | | | | | | | |
| Castro et al. 2018 | -16.2 | 26 | 632 | -4.6 | 30.5 | 321 | 19.7% | -11.60 [-15.50, -7.70] |
| Rape et al. 2018 | -17.3 | 28.3 | 88 | 0.3 | 29.9 | 89 | 13.9% | -17.60 [-26.18, -9.02] |
| Wenzel et al. 2016 | -29.39 | 60.6 | 124 | 10.91 | 59 | 120 | 7.9% | -40.30 [-55.31, -25.29] |
| **Subtotal (95% CI)** | | | **844** | | | **530** | **41.4%** | **-21.18 [-33.97, -8.38]** |
| Heterogeneity: Tau² = 104.38; Chi² = 13.92, df = 2 (P = 0.0009); I² = 86% | | | | | | | | |
| Test for overall effect: Z = 3.24 (P = 0.001) | | | | | | | | |
| **1.6.5 Dupilumab 300 mg q4w** | | | | | | | | |
| Wenzel et al. 2016 | -16.61 | 59.5 | 115 | 10.91 | 59 | 120 | 7.8% | -27.52 [-42.68, -12.36] |
| **Subtotal (95% CI)** | | | **115** | | | **120** | **7.8%** | **-27.52 [-42.68, -12.36]** |
| Heterogeneity: Not applicable | | | | | | | | |
| Test for overall effect: Z = 3.56 (P = 0.0004) | | | | | | | | |
| **Total (95% CI)** | | | **2048** | | | **1326** | **100.0%** | **-19.50 [-24.74, -14.25]** |
| Heterogeneity: Tau² = 32.49; Chi² = 24.32, df = 7 (P = 0.001); I² = 71% | | | | | | | | |
| Test for overall effect: Z = 7.28 (P < 0.00001) | | | | | | | | |
| Test for subgroup differences: Chi² = 1.20, df = 4 (P = 0.88), I² = 0% | | | | | | | | |

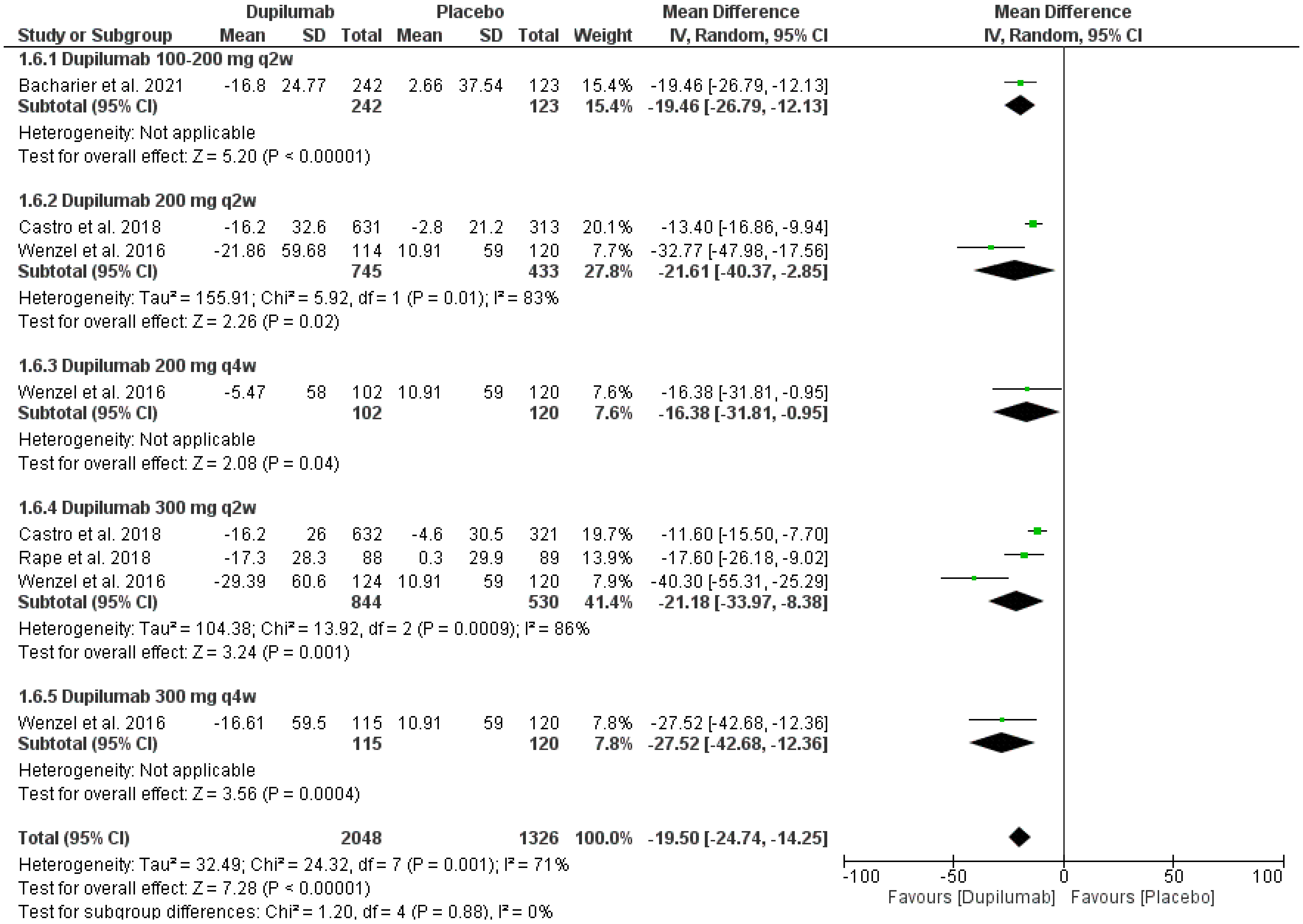

**Supplementary Figures 2B:** A forest plot of the change in FENO (ppb) at week 24 after sensitivity analysis.

| | Dupilumab | | | Placebo | | | | Mean Difference |
|---|---|---|---|---|---|---|---|---|
| **Study or Subgroup** | **Mean** | **SD** | **Total** | **Mean** | **SD** | **Total** | **Weight** | **IV, Random, 95% CI** |
| **1.6.1 Dupilumab 100-200 mg q2w** | | | | | | | | |
| Bacharier et al. 2021 | -16.8 | 24.77 | 242 | 2.66 | 37.54 | 123 | 16.2% | -19.46 [-26.79, -12.13] |
| **Subtotal (95% CI)** | | | **242** | | | **123** | **16.2%** | **-19.46 [-26.79, -12.13]** |
| Heterogeneity: Not applicable | | | | | | | | |
| Test for overall effect: Z = 5.20 (P < 0.00001) | | | | | | | | |
| **1.6.2 Dupilumab 200 mg q2w** | | | | | | | | |
| Castro et al. 2018 | -16.2 | 32.6 | 631 | -2.8 | 21.2 | 313 | 26.5% | -13.40 [-16.86, -9.94] |
| Wenzel et al. 2016 | -21.86 | 59.68 | 114 | 10.91 | 59 | 120 | 6.1% | -32.77 [-47.98, -17.56] |
| **Subtotal (95% CI)** | | | **745** | | | **433** | **32.6%** | **-21.61 [-40.37, -2.85]** |
| Heterogeneity: Tau² = 155.91; Chi² = 5.92, df = 1 (P = 0.01); I² = 83% | | | | | | | | |
| Test for overall effect: Z = 2.26 (P = 0.02) | | | | | | | | |
| **1.6.3 Dupilumab 200 mg q4w** | | | | | | | | |
| Wenzel et al. 2016 | -5.47 | 58 | 102 | 10.91 | 59 | 120 | 6.0% | -16.38 [-31.81, -0.95] |
| **Subtotal (95% CI)** | | | **102** | | | **120** | **6.0%** | **-16.38 [-31.81, -0.95]** |
| Heterogeneity: Not applicable | | | | | | | | |
| Test for overall effect: Z = 2.08 (P = 0.04) | | | | | | | | |
| **1.6.4 Dupilumab 300 mg q2w** | | | | | | | | |
| Castro et al. 2018 | -16.2 | 26 | 632 | -4.6 | 30.5 | 321 | 25.3% | -11.60 [-15.50, -7.70] |
| Rape et al. 2018 | -17.3 | 28.3 | 88 | 0.3 | 29.9 | 89 | 13.7% | -17.60 [-26.18, -9.02] |
| Wenzel et al. 2016 | -29.39 | 60.6 | 124 | 10.91 | 59 | 120 | | Not estimable |
| **Subtotal (95% CI)** | | | **720** | | | **410** | **39.0%** | **-13.34 [-18.67, -8.00]** |
| Heterogeneity: Tau² = 6.44; Chi² = 1.56, df = 1 (P = 0.21); I² = 36% | | | | | | | | |
| Test for overall effect: Z = 4.90 (P < 0.00001) | | | | | | | | |
| **1.6.5 Dupilumab 300 mg q4w** | | | | | | | | |
| Wenzel et al. 2016 | -16.61 | 59.5 | 115 | 10.91 | 59 | 120 | 6.2% | -27.52 [-42.68, -12.36] |
| **Subtotal (95% CI)** | | | **115** | | | **120** | **6.2%** | **-27.52 [-42.68, -12.36]** |
| Heterogeneity: Not applicable | | | | | | | | |
| Test for overall effect: Z = 3.56 (P = 0.0004) | | | | | | | | |
| **Total (95% CI)** | | | **1924** | | | **1206** | **100.0%** | **-16.74 [-20.93, -12.56]** |
| Heterogeneity: Tau² = 14.08; Chi² = 13.20, df = 6 (P = 0.04); I² = 55% | | | | | | | | |
| Test for overall effect: Z = 7.84 (P < 0.00001) | | | | | | | | |
| Test for subgroup differences: Chi² = 4.29, df = 4 (P = 0.37), I² = 6.8% | | | | | | | | |

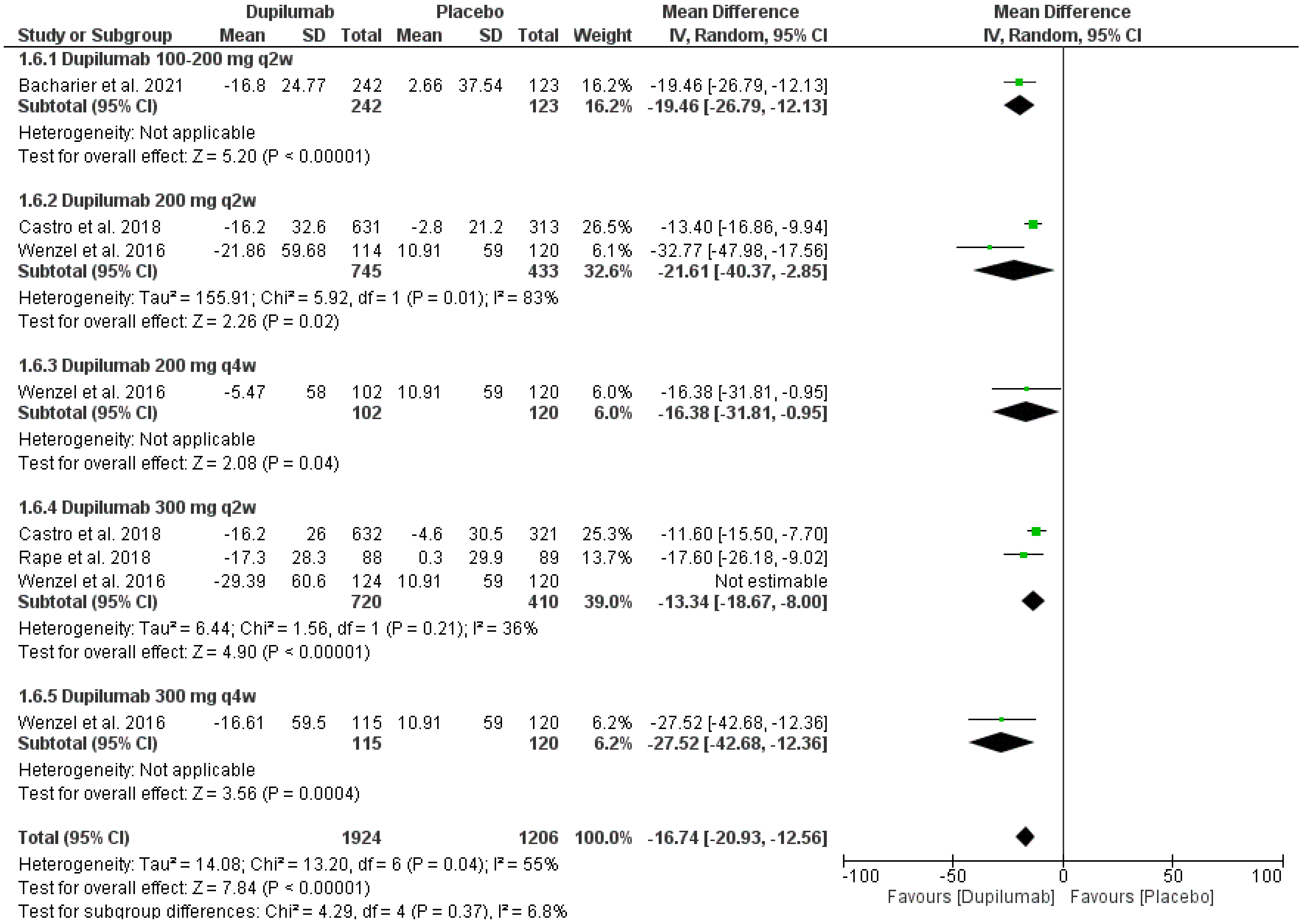

**Supplementary Figure 3:** A forest plot of the change in blood eosinophils (cells/mm$^3$) at week 12.

| Study or Subgroup | Dupilumab Mean | Dupilumab SD | Dupilumab Total | Placebo Mean | Placebo SD | Placebo Total | Weight | Mean Difference IV, Random, 95% CI |
|---|---|---|---|---|---|---|---|---|
| **1.7.1 Dupilumab 100-200 mg q2w** | | | | | | | | |
| Bacharier et al. 2021 | 126.6 | 936 | 255 | 63.8 | 622 | 131 | 5.2% | 62.80 [-93.86, 219.46] |
| **Subtotal (95% CI)** | | | **255** | | | **131** | **5.2%** | **62.80 [-93.86, 219.46]** |
| Heterogeneity: Not applicable | | | | | | | | |
| Test for overall effect: Z = 0.79 (P = 0.43) | | | | | | | | |
| **1.7.2 Dupilumab 200 mg q2w** | | | | | | | | |
| Castro et al. 2018 | 117 | 537 | 631 | -12 | 269 | 313 | 47.9% | 129.00 [77.58, 180.42] |
| **Subtotal (95% CI)** | | | **631** | | | **313** | **47.9%** | **129.00 [77.58, 180.42]** |
| Heterogeneity: Not applicable | | | | | | | | |
| Test for overall effect: Z = 4.92 (P < 0.00001) | | | | | | | | |
| **1.7.3 Dupilumab 300 mg q2w** | | | | | | | | |
| Castro et al. 2018 | 86 | 528 | 632 | -43 | 350 | 321 | 40.1% | 129.00 [72.78, 185.22] |
| Wechsler et al. 2021 | 298.624 | 647 | 56 | -3.517 | 329.5 | 52 | 3.4% | 302.14 [110.47, 493.81] |
| Wenzel et al. 2013 | 171 | 597 | 44 | -2 | 262.2 | 43 | 3.4% | 173.00 [-20.02, 366.02] |
| **Subtotal (95% CI)** | | | **732** | | | **416** | **46.9%** | **168.27 [76.12, 260.41]** |
| Heterogeneity: Tau² = 2633.61; Chi² = 2.97, df = 2 (P = 0.23); I² = 33% | | | | | | | | |
| Test for overall effect: Z = 3.58 (P = 0.0003) | | | | | | | | |
| **Total (95% CI)** | | | **1618** | | | **860** | **100.0%** | **133.05 [97.46, 168.64]** |
| Heterogeneity: Tau² = 0.00; Chi² = 3.97, df = 4 (P = 0.41); I² = 0% | | | | | | | | |
| Test for overall effect: Z = 7.33 (P < 0.00001) | | | | | | | | |
| Test for subgroup differences: Chi² = 1.36, df = 2 (P = 0.51), I² = 0% | | | | | | | | |

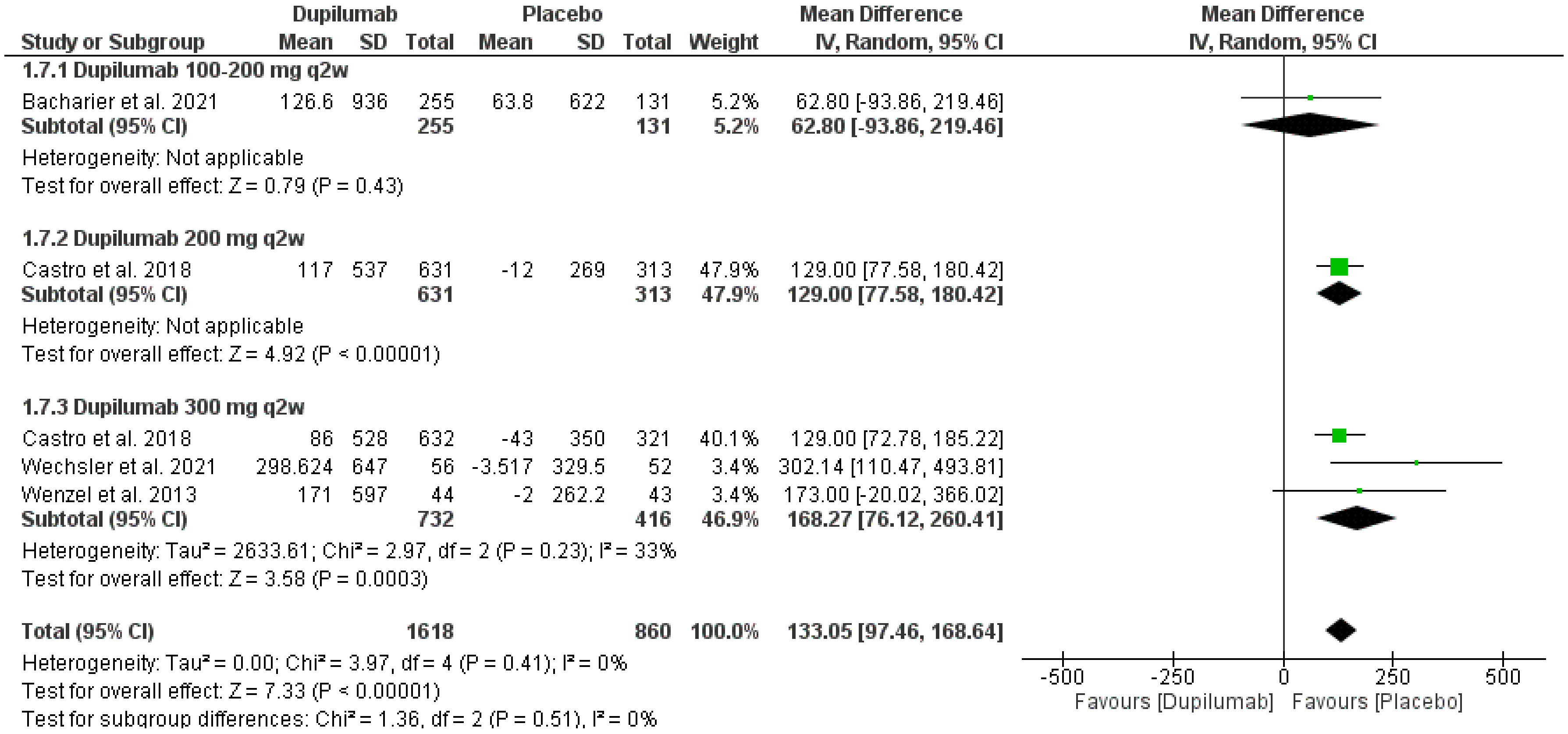

**Supplementary Figure 4:** A forest plot of the change in blood eosinophils (cells/$mm^3$) at week 24.

| Study or Subgroup | Dupilumab Mean | Dupilumab SD | Dupilumab Total | Placebo Mean | Placebo SD | Placebo Total | Weight | Mean Difference IV, Random, 95% CI |
|---|---|---|---|---|---|---|---|---|
| **1.8.1 Dupilumab 100-200 mg q2w** | | | | | | | | |
| Bacharier et al. 2021 | 75.15 | 865 | 250 | -6.16 | 553.1 | 127 | 7.6% | 81.31 [-62.74, 225.36] |
| **Subtotal (95% CI)** | | | **250** | | | **127** | **7.6%** | **81.31 [-62.74, 225.36]** |
| Heterogeneity: Not applicable | | | | | | | | |
| Test for overall effect: Z = 1.11 (P = 0.27) | | | | | | | | |
| **1.8.2 Dupilumab 200 mg q2w** | | | | | | | | |
| Castro et al. 2018 | 84 | 617 | 631 | -23 | 333 | 313 | 42.9% | 107.00 [46.35, 167.65] |
| **Subtotal (95% CI)** | | | **631** | | | **313** | **42.9%** | **107.00 [46.35, 167.65]** |
| Heterogeneity: Not applicable | | | | | | | | |
| Test for overall effect: Z = 3.46 (P = 0.0005) | | | | | | | | |
| **1.8.3 Dupilumab 300 mg q2w** | | | | | | | | |
| Castro et al. 2018 | 54 | 500 | 632 | -32 | 374 | 321 | 49.5% | 86.00 [29.49, 142.51] |
| **Subtotal (95% CI)** | | | **632** | | | **321** | **49.5%** | **86.00 [29.49, 142.51]** |
| Heterogeneity: Not applicable | | | | | | | | |
| Test for overall effect: Z = 2.98 (P = 0.003) | | | | | | | | |
| **Total (95% CI)** | | | **1513** | | | **761** | **100.0%** | **94.66 [54.92, 134.40]** |
| Heterogeneity: Tau² = 0.00; Chi² = 0.28, df = 2 (P = 0.87); I² = 0% | | | | | | | | |
| Test for overall effect: Z = 4.67 (P < 0.00001) | | | | | | | | |
| Test for subgroup differences: Chi² = 0.28, df = 2 (P = 0.87), I² = 0% | | | | | | | | |

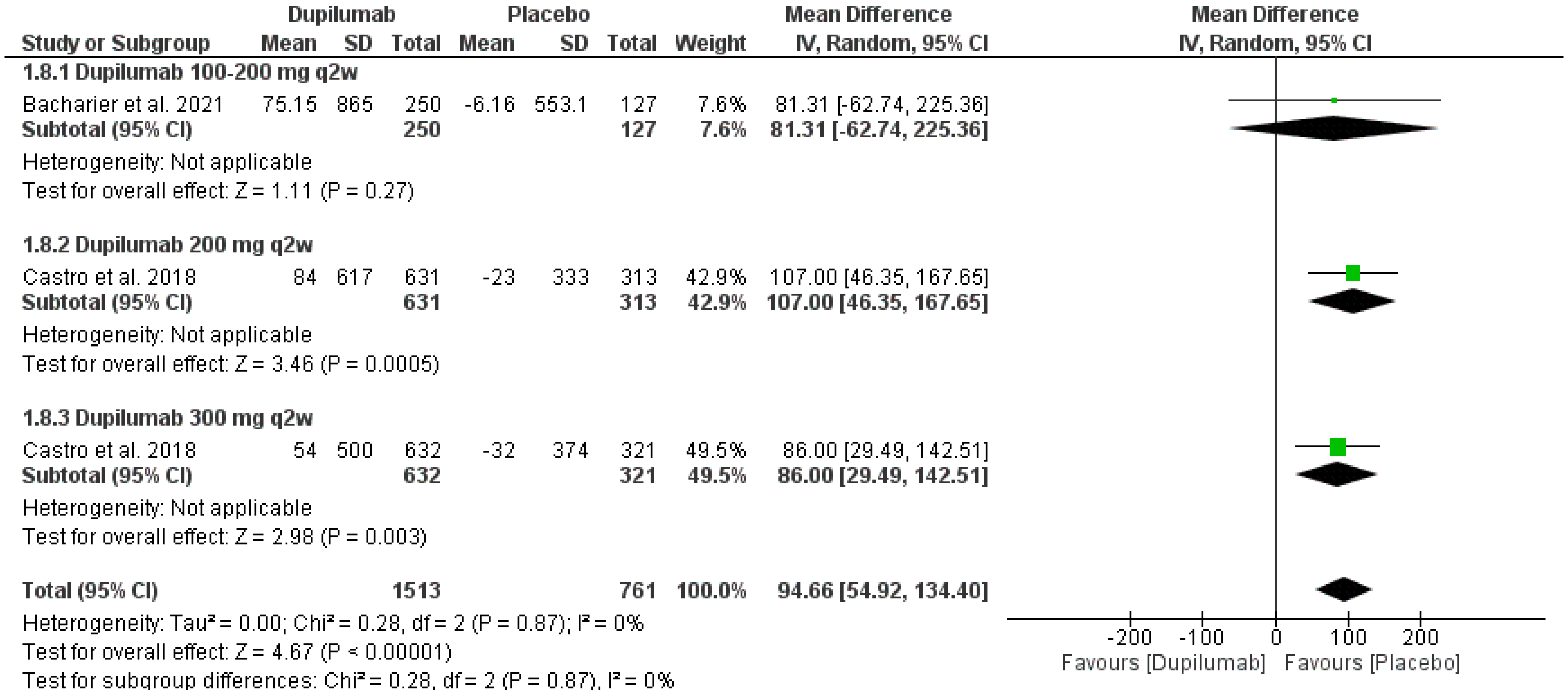

**Supplementary Figure 5:** A forest plot of the change in IgE (IU/mL) at week 12.

| Study or Subgroup | Dupilumab Mean | Dupilumab SD | Dupilumab Total | Placebo Mean | Placebo SD | Placebo Total | Weight | Mean Difference IV, Random, 95% CI |
|---|---|---|---|---|---|---|---|---|
| **1.9.1 Dupilumab 200 mg q2w** | | | | | | | | |
| Castro et al. 2018 | -161.4 | 326.6 | 631 | 11.5 | 306.6 | 313 | 33.6% | -172.90 [-215.36, -130.44] |
| **Subtotal (95% CI)** | | | **631** | | | **313** | **33.6%** | **-172.90 [-215.36, -130.44]** |
| Heterogeneity: Not applicable | | | | | | | | |
| Test for overall effect: Z = 7.98 (P < 0.00001) | | | | | | | | |
| **1.9.2 Dupilumab 300 mg q2w** | | | | | | | | |
| Castro et al. 2018 | -143.4 | 303.6 | 632 | -4.5 | 174.1 | 321 | 56.2% | -138.90 [-169.28, -108.52] |
| Wechsler et al. 2021 | -132.515 | 258 | 63 | -29.141 | 254.4 | 56 | 8.3% | -103.37 [-195.56, -11.19] |
| Wenzel et al. 2013 | -273.32 | 638.2 | 45 | -37.022 | 175.1 | 44 | 1.9% | -236.30 [-429.81, -42.79] |
| **Subtotal (95% CI)** | | | **740** | | | **421** | **66.4%** | **-137.61 [-166.15, -109.08]** |
| Heterogeneity: Tau² = 0.00; Chi² = 1.54, df = 2 (P = 0.46); I² = 0% | | | | | | | | |
| Test for overall effect: Z = 9.45 (P < 0.00001) | | | | | | | | |
| **Total (95% CI)** | | | **1371** | | | **734** | **100.0%** | **-149.27 [-176.39, -122.16]** |
| Heterogeneity: Tau² = 100.24; Chi² = 3.36, df = 3 (P = 0.34); I² = 11% | | | | | | | | |
| Test for overall effect: Z = 10.79 (P < 0.00001) | | | | | | | | |
| Test for subgroup differences: Chi² = 1.83, df = 1 (P = 0.18), I² = 45.3% | | | | | | | | |

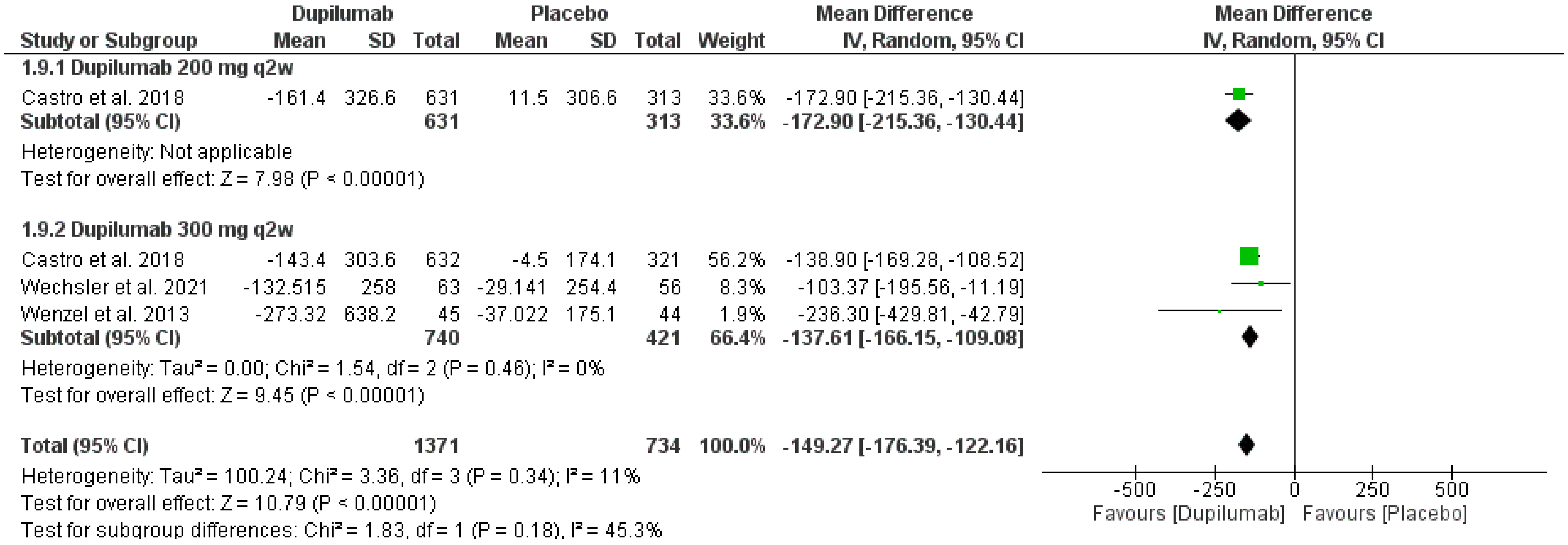

**Supplementary Figure 6:** A forest plot of the change in IgE (IU/mL) at week 24.

| Study or Subgroup | Dupilumab Mean | Dupilumab SD | Dupilumab Total | Placebo Mean | Placebo SD | Placebo Total | Weight | Mean Difference IV, Random, 95% CI |
|---|---|---|---|---|---|---|---|---|
| **1.10.1 Dupilumab 100-200 mg q2w** | | | | | | | | |
| Bacharier et al. 2021 | -431.75 | 1,292 | 265 | -15.91 | 1,580.7 | 130 | 13.4% | -415.84 [-728.94, -102.74] |
| **Subtotal (95% CI)** | | | **265** | | | **130** | **13.4%** | **-415.84 [-728.94, -102.74]** |
| Heterogeneity: Not applicable | | | | | | | | |
| Test for overall effect: Z = 2.60 (P = 0.009) | | | | | | | | |
| **1.10.2 Dupilumab 200 mg q2w** | | | | | | | | |
| Castro et al. 2018 | -245.6 | 461.1 | 631 | 10.5 | 303.7 | 313 | 28.6% | -256.10 [-305.36, -206.84] |
| **Subtotal (95% CI)** | | | **631** | | | **313** | **28.6%** | **-256.10 [-305.36, -206.84]** |
| Heterogeneity: Not applicable | | | | | | | | |
| Test for overall effect: Z = 10.19 (P < 0.00001) | | | | | | | | |
| **1.10.3 Dupilumab 300 mg q2w** | | | | | | | | |
| Bachert et al. 2020 | -36.81 | 120.21 | 297 | 2.72 | 49.25 | 150 | 29.4% | -39.53 [-55.31, -23.75] |
| Castro et al. 2018 | -210.4 | 367.6 | 632 | 33.5 | 372.2 | 321 | 28.6% | -243.90 [-293.69, -194.11] |
| **Subtotal (95% CI)** | | | **929** | | | **471** | **58.0%** | **-140.29 [-340.55, 59.96]** |
| Heterogeneity: $Tau^2 = 20528.45$; $Chi^2 = 58.81$, df = 1 ($P < 0.00001$); $I^2 = 98\%$ | | | | | | | | |
| Test for overall effect: Z = 1.37 (P = 0.17) | | | | | | | | |
| **Total (95% CI)** | | | **1825** | | | **914** | **100.0%** | **-210.28 [-365.02, -55.55]** |
| Heterogeneity: $Tau^2 = 21137.28$; $Chi^2 = 120.08$, df = 3 ($P < 0.00001$); $I^2 = 98\%$ | | | | | | | | |
| Test for overall effect: Z = 2.66 (P = 0.008) | | | | | | | | |
| Test for subgroup differences: $Chi^2 = 2.27$, df = 2 (P = 0.32), $I^2 = 11.9\%$ | | | | | | | | |

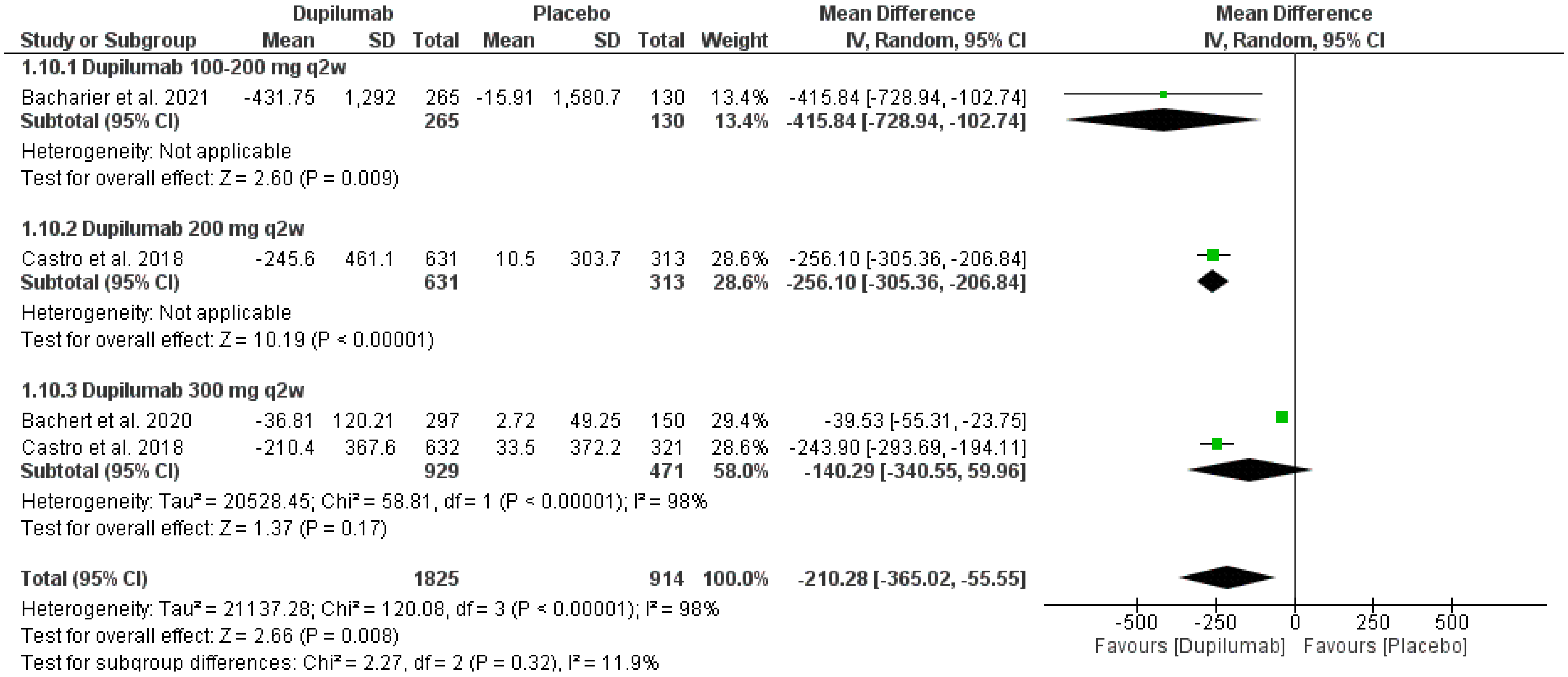

**Supplementary Figure 7:** A forest plot of any and treatment-emergent adverse events.

| Study or Subgroup | Dupilumab Events | Dupilumab Total | Placebo Events | Placebo Total | Weight | Risk Ratio M-H, Random, 95% CI |
|---|---|---|---|---|---|---|
| **1.11.1 Any adverse events** | | | | | | |
| Bacharier et al. 2021 | 225 | 271 | 107 | 134 | 14.5% | 1.04 [0.94, 1.15] |
| Castro et al. 2018 | 1023 | 1263 | 527 | 634 | 75.4% | 0.97 [0.93, 1.02] |
| Rape et al. 2018 | 64 | 103 | 69 | 107 | 3.4% | 0.96 [0.78, 1.18] |
| Wechsler et al. 2021 | 49 | 74 | 52 | 74 | 3.0% | 0.94 [0.76, 1.17] |
| Wenzel et al. 2013 | 42 | 52 | 40 | 52 | 3.7% | 1.05 [0.86, 1.28] |
| **Subtotal (95% CI)** | | **1763** | | **1001** | **100.0%** | **0.98 [0.95, 1.02]** |
| Total events | 1403 | | 795 | | | |
| Heterogeneity: Tau² = 0.00; Chi² = 1.93, df = 4 (P = 0.75); I² = 0% | | | | | | |
| Test for overall effect: Z = 0.78 (P = 0.44) | | | | | | |
| **1.11.2 Any treatment-emergent adverse events** | | | | | | |
| Laidlaw et al. 2021 | 68 | 260 | 34 | 167 | 7.0% | 1.28 [0.89, 1.85] |
| Wechsler et al. 2021 | 3 | 74 | 3 | 74 | 0.4% | 1.00 [0.21, 4.79] |
| Wenzel et al. 2016 | 483 | 611 | 118 | 158 | 92.7% | 1.06 [0.96, 1.17] |
| **Subtotal (95% CI)** | | **945** | | **399** | **100.0%** | **1.07 [0.97, 1.18]** |
| Total events | 554 | | 155 | | | |
| Heterogeneity: Tau² = 0.00; Chi² = 1.26, df = 2 (P = 0.53); I² = 0% | | | | | | |
| Test for overall effect: Z = 1.43 (P = 0.15) | | | | | | |

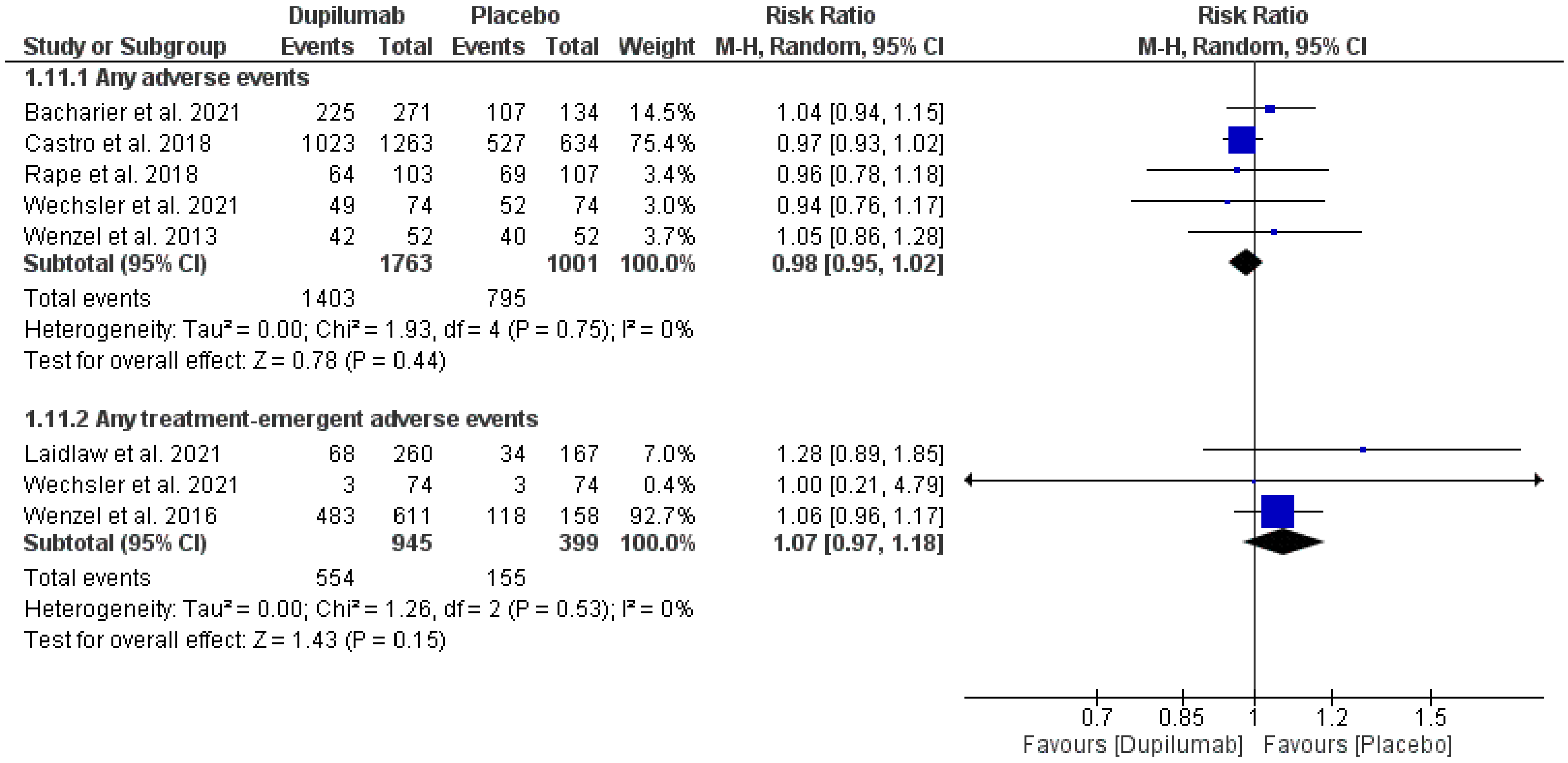

**Supplementary Figure 8:** A forest plot of any and treatment-emergent adverse events leading to permanent discontinuation.

| | Dupilumab | | Placebo | | | Risk Ratio |
|---|---|---|---|---|---|---|
| **Study or Subgroup** | **Events** | **Total** | **Events** | **Total** | **Weight** | **M-H, Random, 95% CI** |
| **1.12.1 Any adverse events leading to permanent discontinuation** | | | | | | |
| Bacharier et al. 2021 | 5 | 271 | 2 | 134 | 5.8% | 1.24 [0.24, 6.29] |
| Castro et al. 2018 | 63 | 1263 | 29 | 634 | 82.9% | 1.09 [0.71, 1.68] |
| Rape et al. 2018 | 1 | 103 | 4 | 107 | 3.2% | 0.26 [0.03, 2.28] |
| Wechsler et al. 2021 | 0 | 74 | 3 | 74 | 1.8% | 0.14 [0.01, 2.72] |
| Wenzel et al. 2013 | 3 | 52 | 3 | 52 | 6.3% | 1.00 [0.21, 4.73] |
| **Subtotal (95% CI)** | | **1763** | | **1001** | **100.0%** | **1.01 [0.68, 1.49]** |
| Total events | 72 | | 41 | | | |
| Heterogeneity: Tau² = 0.00; Chi² = 3.42, df = 4 (P = 0.49); I² = 0% | | | | | | |
| Test for overall effect: Z = 0.03 (P = 0.98) | | | | | | |
| **1.12.3 Any treatment-emergent adverse events leading to permanent discontinuation** | | | | | | |
| Laidlaw et al. 2021 | 13 | 260 | 7 | 167 | 52.2% | 1.19 [0.49, 2.93] |
| Wenzel et al. 2016 | 27 | 611 | 5 | 158 | 47.8% | 1.40 [0.55, 3.57] |
| **Subtotal (95% CI)** | | **871** | | **325** | **100.0%** | **1.29 [0.67, 2.46]** |
| Total events | 40 | | 12 | | | |
| Heterogeneity: Tau² = 0.00; Chi² = 0.06, df = 1 (P = 0.81); I² = 0% | | | | | | |
| Test for overall effect: Z = 0.76 (P = 0.45) | | | | | | |

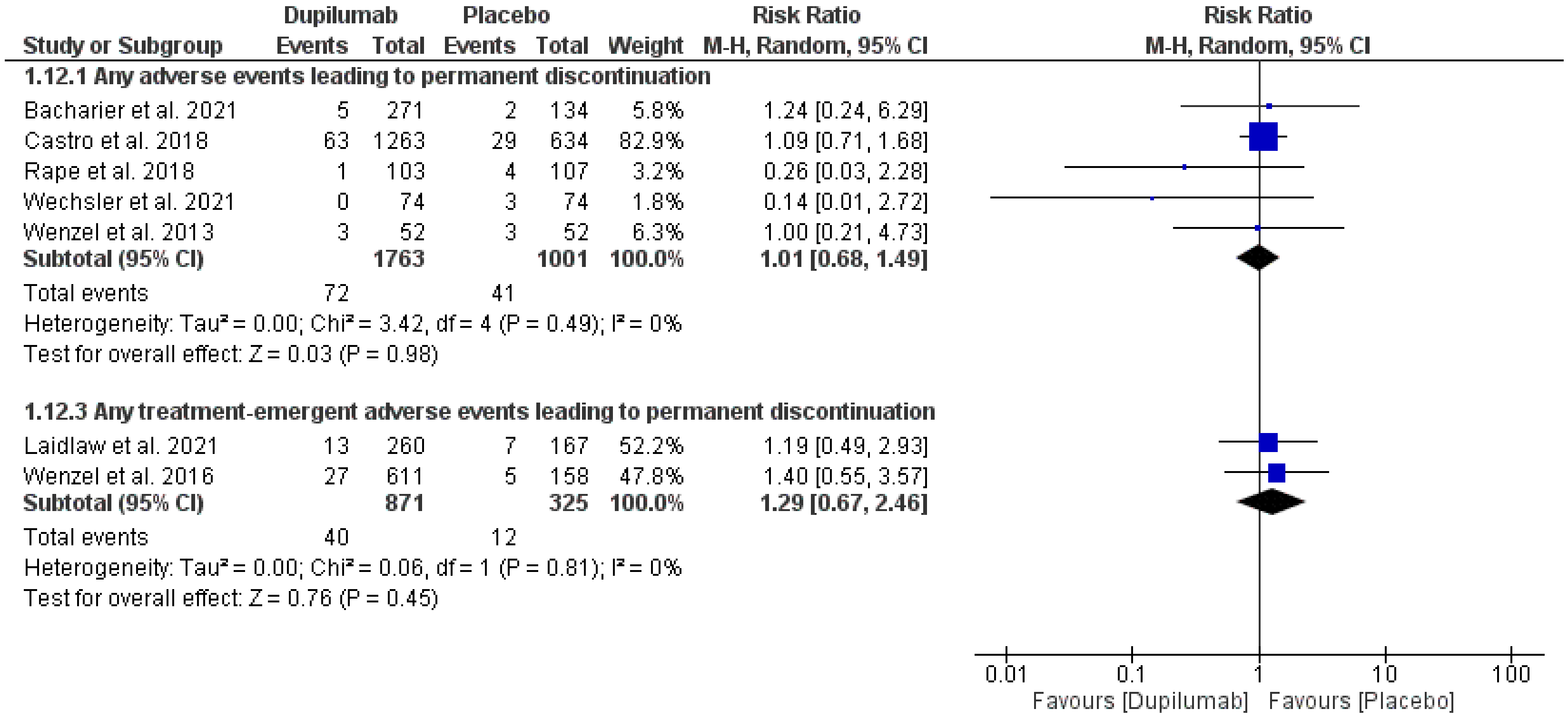

**Supplementary Figure 9:** A forest plot of serious and treatment-emergent adverse events.

| Study or Subgroup | Dupilumab Events | Dupilumab Total | Placebo Events | Placebo Total | Weight | Risk Ratio M-H, Random, 95% CI |
|---|---|---|---|---|---|---|
| **1.13.1 Serious adverse events** | | | | | | |
| Bacharier et al. 2021 | 13 | 271 | 6 | 134 | 9.1% | 1.07 [0.42, 2.76] |
| Castro et al. 2018 | 104 | 1263 | 53 | 634 | 81.1% | 0.99 [0.72, 1.35] |
| Rape et al. 2018 | 9 | 103 | 6 | 107 | 8.2% | 1.56 [0.58, 4.22] |
| Wenzel et al. 2013 | 1 | 52 | 3 | 52 | 1.6% | 0.33 [0.04, 3.10] |
| **Subtotal (95% CI)** | | **1689** | | **927** | **100.0%** | **1.01 [0.76, 1.35]** |
| Total events | 127 | | 68 | | | |
| Heterogeneity: Tau² = 0.00; Chi² = 1.71, df = 3 (P = 0.63); I² = 0% | | | | | | |
| Test for overall effect: Z = 0.09 (P = 0.93) | | | | | | |
| **1.13.2 Serious treatment-emergent adverse events** | | | | | | |
| Laidlaw et al. 2021 | 13 | 260 | 7 | 167 | 37.4% | 1.19 [0.49, 2.93] |
| Wenzel et al. 2016 | 45 | 611 | 9 | 158 | 62.6% | 1.29 [0.65, 2.59] |
| **Subtotal (95% CI)** | | **871** | | **325** | **100.0%** | **1.25 [0.72, 2.17]** |
| Total events | 58 | | 16 | | | |
| Heterogeneity: Tau² = 0.00; Chi² = 0.02, df = 1 (P = 0.89); I² = 0% | | | | | | |
| Test for overall effect: Z = 0.81 (P = 0.42) | | | | | | |

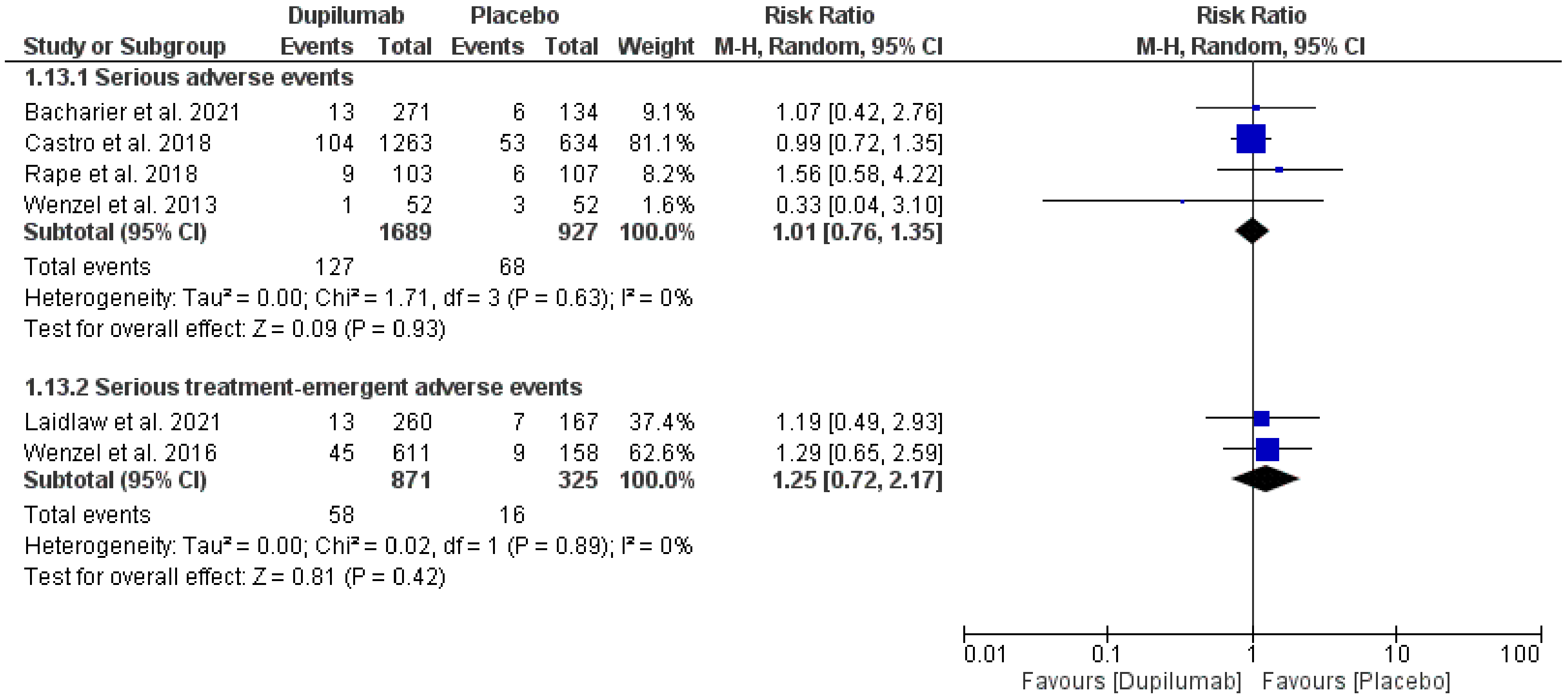

**Supplementary Figure 10:** A forest plot of any adverse events leading to death and any treatment-emergent adverse events leading to death.

| Study or Subgroup | Dupilumab Events | Dupilumab Total | Placebo Events | Placebo Total | Weight | Risk Ratio M-H, Random, 95% CI |
|---|---|---|---|---|---|---|
| **1.14.1 Any adverse events leading to death** | | | | | | |
| Castro et al. 2018 | 5 | 1263 | 3 | 634 | 83.3% | 0.84 [0.20, 3.49] |
| Rape et al. 2018 | 0 | 103 | 0 | 107 | | Not estimable |
| Wechsler et al. 2021 | 1 | 74 | 0 | 74 | 16.7% | 3.00 [0.12, 72.47] |
| Wenzel et al. 2013 | 0 | 52 | 0 | 52 | | Not estimable |
| **Subtotal (95% CI)** | | **1492** | | **867** | **100.0%** | **1.04 [0.28, 3.81]** |
| Total events | 6 | | 3 | | | |
| Heterogeneity: Tau² = 0.00; Chi² = 0.52, df = 1 (P = 0.47); I² = 0% | | | | | | |
| Test for overall effect: Z = 0.05 (P = 0.96) | | | | | | |
| **1.14.2 Any treatment-emergent adverse events leading to death** | | | | | | |
| Laidlaw et al. 2021 | 0 | 260 | 0 | 167 | | Not estimable |
| Wenzel et al. 2016 | 2 | 611 | 0 | 158 | 100.0% | 1.30 [0.06, 26.92] |
| **Subtotal (95% CI)** | | **871** | | **325** | **100.0%** | **1.30 [0.06, 26.92]** |
| Total events | 2 | | 0 | | | |
| Heterogeneity: Not applicable | | | | | | |
| Test for overall effect: Z = 0.17 (P = 0.87) | | | | | | |

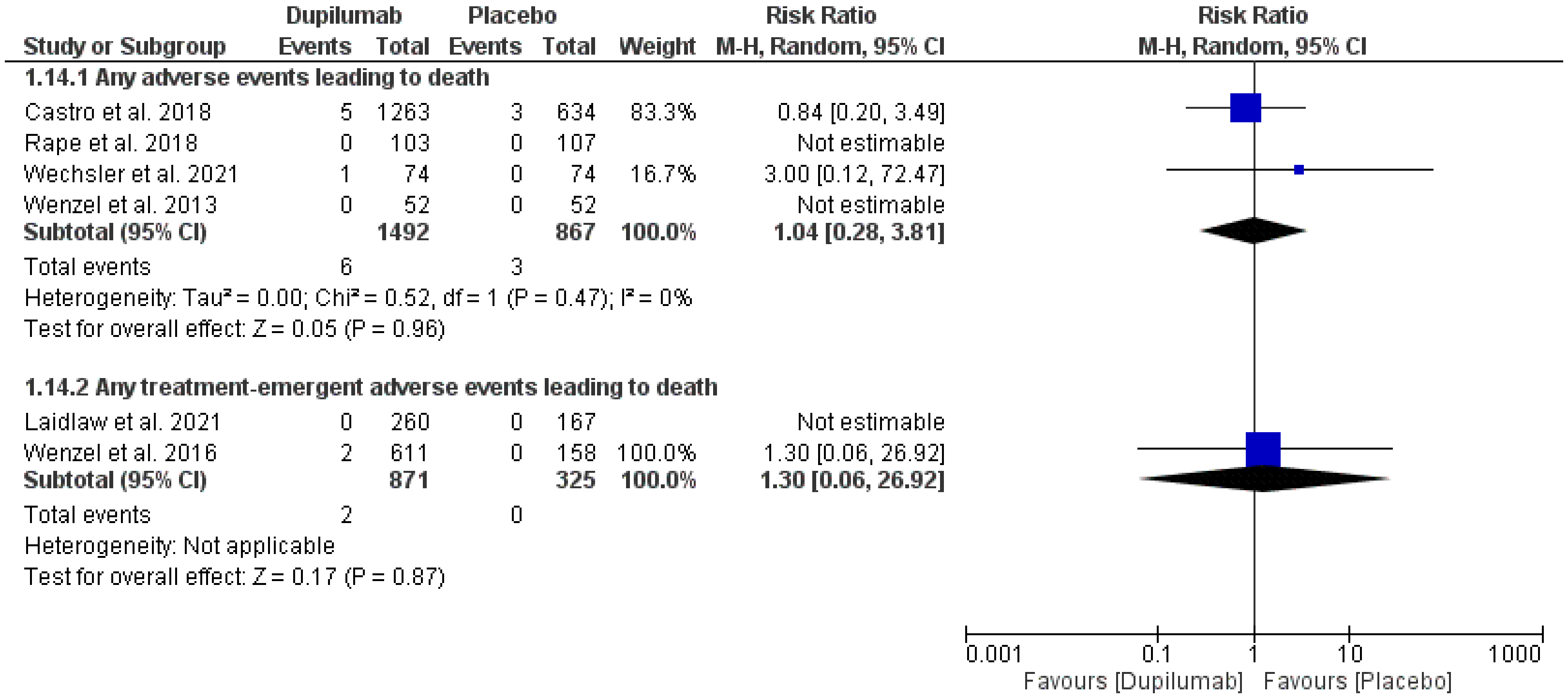

**Supplementary Figure 11:** A forest plot of upper respiratory tract infection, viral upper respiratory tract infection, and influenza adverse events.

| Study or Subgroup | Dupilumab Events | Dupilumab Total | Placebo Events | Placebo Total | Weight | Risk Ratio M-H, Random, 95% CI |
|---|---|---|---|---|---|---|
| **1.15.1 Upper respiratory tract infection** | | | | | | |
| Bacharier et al. 2021 | 35 | 271 | 18 | 134 | 12.1% | 0.96 [0.57, 1.63] |
| Castro et al. 2018 | 146 | 1263 | 86 | 634 | 54.8% | 0.85 [0.66, 1.09] |
| Laidlaw et al. 2021 | 9 | 260 | 10 | 167 | 4.4% | 0.58 [0.24, 1.39] |
| Wechsler et al. 2021 | 4 | 74 | 7 | 74 | 2.4% | 0.57 [0.17, 1.87] |
| Wenzel et al. 2013 | 7 | 52 | 9 | 52 | 4.1% | 0.78 [0.31, 1.93] |
| Wenzel et al. 2016 | 83 | 611 | 28 | 158 | 22.2% | 0.77 [0.52, 1.13] |
| **Subtotal (95% CI)** | | **2531** | | **1219** | **100.0%** | **0.82 [0.68, 0.99]** |
| Total events | 284 | | 158 | | | |
| Heterogeneity: Tau² = 0.00; Chi² = 1.53, df = 5 (P = 0.91); I² = 0% | | | | | | |
| Test for overall effect: Z = 2.12 (P = 0.03) | | | | | | |
| **1.15.2 Viral upper respiratory tract infection** | | | | | | |
| Bacharier et al. 2021 | 33 | 271 | 13 | 134 | 24.5% | 1.26 [0.68, 2.30] |
| Castro et al. 2018 | 230 | 1263 | 124 | 634 | 49.0% | 0.93 [0.77, 1.13] |
| Rape et al. 2018 | 9 | 103 | 19 | 107 | 19.1% | 0.49 [0.23, 1.04] |
| Wechsler et al. 2021 | 2 | 74 | 5 | 74 | 5.6% | 0.40 [0.08, 2.00] |
| Wenzel et al. 2013 | 3 | 52 | 0 | 52 | 1.8% | 7.00 [0.37, 132.23] |
| **Subtotal (95% CI)** | | **1763** | | **1001** | **100.0%** | **0.88 [0.59, 1.31]** |
| Total events | 277 | | 161 | | | |
| Heterogeneity: Tau² = 0.08; Chi² = 6.59, df = 4 (P = 0.16); I² = 39% | | | | | | |
| Test for overall effect: Z = 0.64 (P = 0.52) | | | | | | |
| **1.15.3 Influenza** | | | | | | |
| Castro et al. 2018 | 74 | 1263 | 51 | 634 | 52.3% | 0.73 [0.52, 1.03] |
| Rape et al. 2018 | 3 | 103 | 6 | 107 | 18.2% | 0.52 [0.13, 2.02] |
| Wenzel et al. 2016 | 38 | 611 | 5 | 158 | 29.4% | 1.97 [0.79, 4.91] |
| **Subtotal (95% CI)** | | **1977** | | **899** | **100.0%** | **0.92 [0.46, 1.84]** |
| Total events | 115 | | 62 | | | |
| Heterogeneity: Tau² = 0.21; Chi² = 4.43, df = 2 (P = 0.11); I² = 55% | | | | | | |
| Test for overall effect: Z = 0.24 (P = 0.81) | | | | | | |

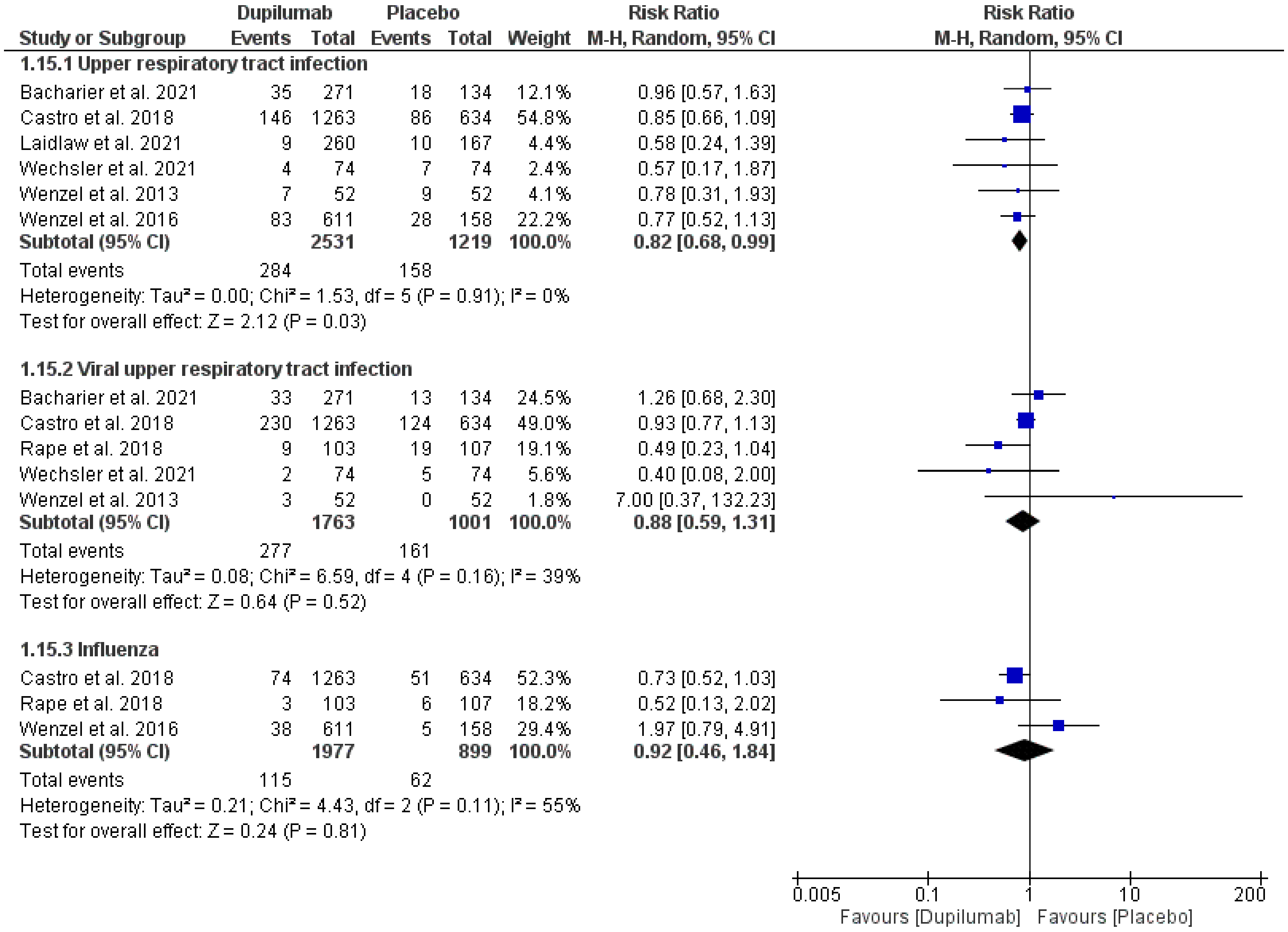

**Supplementary Figure 12:** A forest plot of nasopharyngitis, sinusitis, and bronchitis adverse events.

| Study or Subgroup | Dupilumab Events | Dupilumab Total | Placebo Events | Placebo Total | Weight | Risk Ratio M-H, Random, 95% CI |
|---|---|---|---|---|---|---|
| **1.16.1 Nasopharyngitis** | | | | | | |
| Bacharier et al. 2021 | 50 | 271 | 29 | 134 | 39.0% | 0.85 [0.57, 1.28] |
| Laidlaw et al. 2021 | 33 | 260 | 24 | 167 | 27.2% | 0.88 [0.54, 1.44] |
| Wechsler et al. 2021 | 9 | 74 | 9 | 74 | 8.7% | 1.00 [0.42, 2.38] |
| Wenzel et al. 2013 | 7 | 52 | 2 | 52 | 2.8% | 3.50 [0.76, 16.06] |
| Wenzel et al. 2016 | 59 | 611 | 15 | 158 | 22.3% | 1.02 [0.59, 1.74] |
| **Subtotal (95% CI)** | | **1268** | | **585** | **100.0%** | **0.94 [0.73, 1.22]** |
| Total events | 158 | | 79 | | | |
| Heterogeneity: Tau² = 0.00; Chi² = 3.27, df = 4 (P = 0.51); I² = 0% | | | | | | |
| Test for overall effect: Z = 0.44 (P = 0.66) | | | | | | |
| **1.16.2 Sinusitis** | | | | | | |
| Bacharier et al. 2021 | 9 | 271 | 7 | 134 | 27.0% | 0.64 [0.24, 1.67] |
| Rape et al. 2018 | 7 | 103 | 4 | 107 | 19.0% | 1.82 [0.55, 6.03] |
| Wenzel et al. 2013 | 1 | 52 | 5 | 52 | 6.8% | 0.20 [0.02, 1.65] |
| Wenzel et al. 2016 | 36 | 611 | 11 | 158 | 47.1% | 0.85 [0.44, 1.62] |
| **Subtotal (95% CI)** | | **1037** | | **451** | **100.0%** | **0.82 [0.47, 1.45]** |
| Total events | 53 | | 27 | | | |
| Heterogeneity: Tau² = 0.07; Chi² = 3.69, df = 3 (P = 0.30); I² = 19% | | | | | | |
| Test for overall effect: Z = 0.68 (P = 0.50) | | | | | | |
| **1.16.4 Bronchitis** | | | | | | |
| Bacharier et al. 2021 | 17 | 271 | 14 | 134 | 9.6% | 0.60 [0.31, 1.18] |
| Castro et al. 2018 | 144 | 1263 | 89 | 634 | 72.0% | 0.81 [0.63, 1.04] |
| Rape et al. 2018 | 7 | 103 | 6 | 107 | 3.9% | 1.21 [0.42, 3.49] |
| Wenzel et al. 2016 | 51 | 611 | 15 | 158 | 14.5% | 0.88 [0.51, 1.52] |
| **Subtotal (95% CI)** | | **2248** | | **1033** | **100.0%** | **0.81 [0.66, 1.00]** |
| Total events | 219 | | 124 | | | |
| Heterogeneity: Tau² = 0.00; Chi² = 1.40, df = 3 (P = 0.71); I² = 0% | | | | | | |
| Test for overall effect: Z = 1.97 (P = 0.05) | | | | | | |

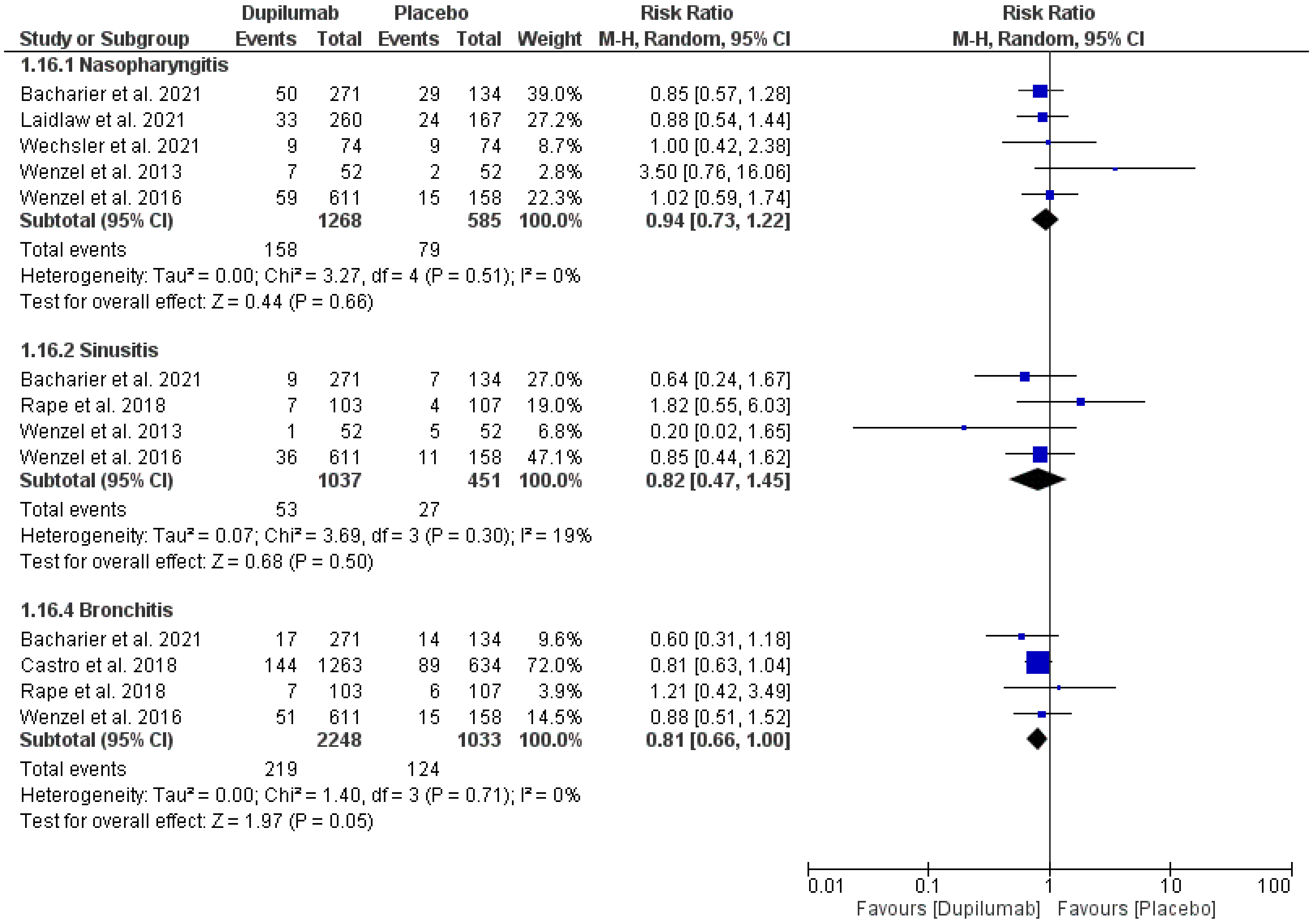

**Supplementary Figure 13:** A forest plot of injection site reaction, eosinophilia, and headache adverse events.

| | Dupilumab | | Placebo | | | Risk Ratio |
|---|---|---|---|---|---|---|
| **Study or Subgroup** | **Events** | **Total** | **Events** | **Total** | **Weight** | **M-H, Random, 95% CI** |
| **1.17.1 Injection-site reaction** | | | | | | |
| Bacharier et al. 2021 | 80 | 271 | 23 | 134 | 19.8% | 1.72 [1.14, 2.60] |
| Castro et al. 2018 | 212 | 1263 | 50 | 634 | 29.0% | 2.13 [1.59, 2.85] |
| Laidlaw et al. 2021 | 60 | 260 | 33 | 167 | 22.2% | 1.17 [0.80, 1.70] |
| Rape et al. 2018 | 9 | 103 | 4 | 107 | 3.8% | 2.34 [0.74, 7.35] |
| Wechsler et al. 2021 | 4 | 74 | 4 | 74 | 2.8% | 1.00 [0.26, 3.85] |
| Weinstein et al. 2018 | 60 | 249 | 19 | 140 | 16.7% | 1.78 [1.11, 2.85] |
| Wenzel et al. 2013 | 15 | 52 | 5 | 52 | 5.5% | 3.00 [1.18, 7.65] |
| **Subtotal (95% CI)** | | **2272** | | **1308** | **100.0%** | **1.73 [1.37, 2.19]** |
| Total events | 440 | | 138 | | | |
| Heterogeneity: Tau² = 0.03; Chi² = 8.38, df = 6 (P = 0.21); I² = 28% | | | | | | |
| Test for overall effect: Z = 4.64 (P < 0.00001) | | | | | | |
| **1.17.2 Eosinophilia** | | | | | | |
| Bacharier et al. 2021 | 16 | 271 | 1 | 134 | 50.0% | 7.91 [1.06, 59.03] |
| Rape et al. 2018 | 14 | 103 | 1 | 107 | 50.0% | 14.54 [1.95, 108.61] |
| **Subtotal (95% CI)** | | **374** | | **241** | **100.0%** | **10.73 [2.59, 44.43]** |
| Total events | 30 | | 2 | | | |
| Heterogeneity: Tau² = 0.00; Chi² = 0.18, df = 1 (P = 0.67); I² = 0% | | | | | | |
| Test for overall effect: Z = 3.27 (P = 0.001) | | | | | | |
| **1.17.3 Headache** | | | | | | |
| Bacharier et al. 2021 | 19 | 271 | 10 | 134 | 9.4% | 0.94 [0.45, 1.96] |
| Castro et al. 2018 | 86 | 1263 | 51 | 634 | 45.9% | 0.85 [0.61, 1.18] |
| Laidlaw et al. 2021 | 20 | 260 | 16 | 167 | 12.9% | 0.80 [0.43, 1.50] |
| Wechsler et al. 2021 | 10 | 74 | 7 | 74 | 6.1% | 1.43 [0.57, 3.55] |
| Wenzel et al. 2013 | 6 | 52 | 3 | 52 | 2.9% | 2.00 [0.53, 7.57] |
| Wenzel et al. 2016 | 62 | 611 | 20 | 158 | 22.8% | 0.80 [0.50, 1.29] |
| **Subtotal (95% CI)** | | **2531** | | **1219** | **100.0%** | **0.89 [0.71, 1.11]** |
| Total events | 203 | | 107 | | | |
| Heterogeneity: Tau² = 0.00; Chi² = 2.86, df = 5 (P = 0.72); I² = 0% | | | | | | |
| Test for overall effect: Z = 1.04 (P = 0.30) | | | | | | |

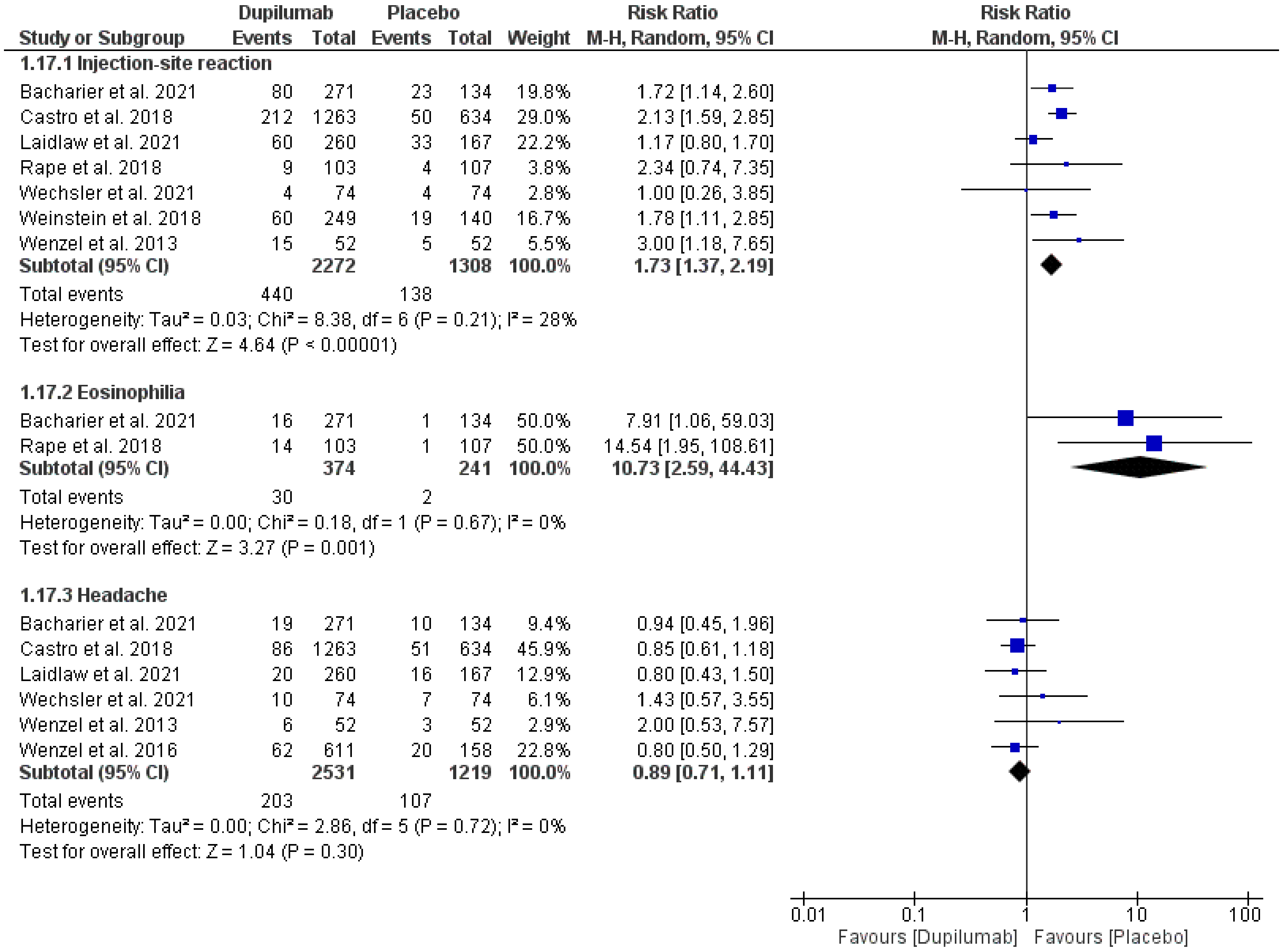

**Supplementary Figure 14:** A forest plot of allergic rhinitis, cough, urinary tract infection, back pain, and erythema adverse events.

| Study or Subgroup | Dupilumab Events | Dupilumab Total | Placebo Events | Placebo Total | Weight | Risk Ratio M-H, Random, 95% CI |
|---|---|---|---|---|---|---|
| **1.18.1 Allergic rhinitis** | | | | | | |
| Bacharier et al. 2021 | 16 | 271 | 16 | 134 | 40.5% | 0.49 [0.26, 0.96] |
| Castro et al. 2018 | 39 | 1263 | 31 | 634 | 50.9% | 0.63 [0.40, 1.00] |
| Wechsler et al. 2021 | 5 | 74 | 1 | 74 | 8.6% | 5.00 [0.60, 41.77] |
| **Subtotal (95% CI)** | | **1608** | | **842** | **100.0%** | **0.68 [0.35, 1.33]** |
| Total events | 60 | | 48 | | | |
| Heterogeneity: Tau² = 0.17; Chi² = 4.24, df = 2 (P = 0.12); I² = 53% | | | | | | |
| Test for overall effect: Z = 1.12 (P = 0.26) | | | | | | |
| **1.18.2 Cough** | | | | | | |
| Bacharier et al. 2021 | 15 | 271 | 9 | 134 | 74.5% | 0.82 [0.37, 1.83] |
| Wechsler et al. 2021 | 1 | 74 | 5 | 74 | 25.5% | 0.20 [0.02, 1.67] |
| **Subtotal (95% CI)** | | **345** | | **208** | **100.0%** | **0.57 [0.17, 1.96]** |
| Total events | 16 | | 14 | | | |
| Heterogeneity: Tau² = 0.36; Chi² = 1.54, df = 1 (P = 0.22); I² = 35% | | | | | | |
| Test for overall effect: Z = 0.89 (P = 0.37) | | | | | | |
| **1.18.3 Urinary tract infection** | | | | | | |
| Castro et al. 2018 | 36 | 1263 | 29 | 634 | 93.1% | 0.62 [0.39, 1.01] |
| Wechsler et al. 2021 | 3 | 74 | 2 | 74 | 6.9% | 1.50 [0.26, 8.72] |
| **Subtotal (95% CI)** | | **1337** | | **708** | **100.0%** | **0.66 [0.42, 1.05]** |
| Total events | 39 | | 31 | | | |
| Heterogeneity: Tau² = 0.00; Chi² = 0.89, df = 1 (P = 0.35); I² = 0% | | | | | | |
| Test for overall effect: Z = 1.75 (P = 0.08) | | | | | | |
| **1.18.4 Back pain** | | | | | | |
| Castro et al. 2018 | 55 | 1263 | 23 | 634 | 95.7% | 1.20 [0.74, 1.93] |
| Wechsler et al. 2021 | 3 | 74 | 1 | 74 | 4.3% | 3.00 [0.32, 28.18] |
| **Subtotal (95% CI)** | | **1337** | | **708** | **100.0%** | **1.25 [0.78, 1.99]** |
| Total events | 58 | | 24 | | | |
| Heterogeneity: Tau² = 0.00; Chi² = 0.62, df = 1 (P = 0.43); I² = 0% | | | | | | |
| Test for overall effect: Z = 0.93 (P = 0.35) | | | | | | |
| **1.18.5 Erythema** | | | | | | |
| Bacharier et al. 2021 | 35 | 271 | 13 | 134 | 54.8% | 1.33 [0.73, 2.43] |
| Laidlaw et al. 2021 | 19 | 260 | 14 | 167 | 45.2% | 0.87 [0.45, 1.69] |
| **Subtotal (95% CI)** | | **531** | | **301** | **100.0%** | **1.10 [0.70, 1.72]** |
| Total events | 54 | | 27 | | | |
| Heterogeneity: Tau² = 0.00; Chi² = 0.86, df = 1 (P = 0.35); I² = 0% | | | | | | |
| Test for overall effect: Z = 0.42 (P = 0.68) | | | | | | |

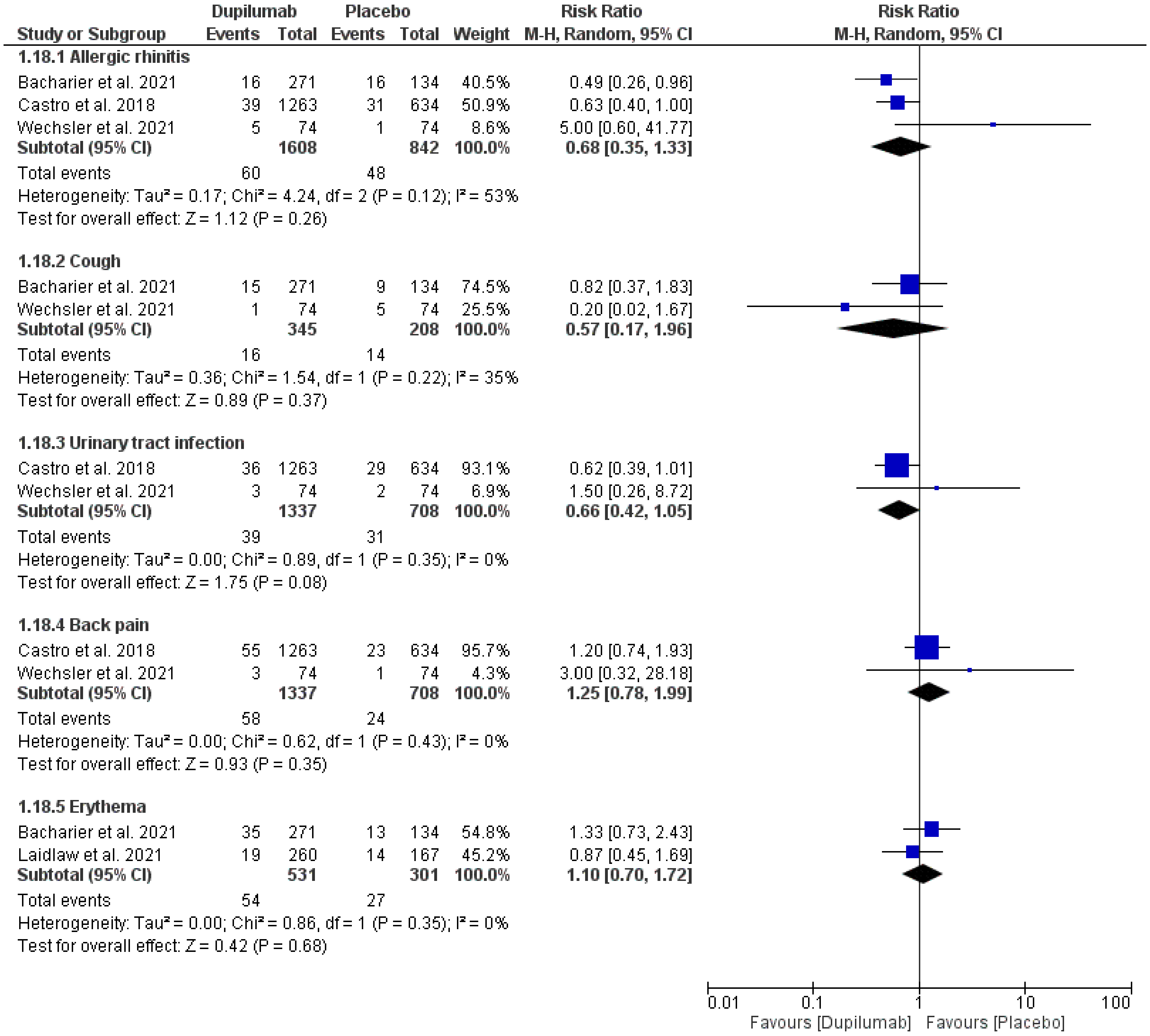

Supplement: Supplementary file 1 [file Presentation1.pdf]
